# Soil fertility determines whether ectomycorrhizal fungi accelerate or decelerate decomposition in a temperate forest

**DOI:** 10.1111/nph.18930

**Published:** 2023-04-21

**Authors:** Mathias Mayer, Bradley Matthews, Hans Sandén, Klaus Katzensteiner, Frank Hagedorn, Markus Gorfer, Harald Berger, Torsten W. Berger, Douglas L. Godbold, Boris Rewald

**Affiliations:** 1 Forest Soils and Biogeochemistry, Swiss Federal Institute for Forest, Snow and Landscape Research (WSL), Zürcherstrasse 111, Birmensdorf 8903, Switzerland; 2 Forest Ecology, Institute of Terrestrial Ecosystems (ITES), ETH Zurich, Universitätsstrasse 16, Zürich 8092, Switzerland; 3 Department of Forest and Soil Sciences, Institute of Forest Ecology, University of Natural Resources and Life Sciences (BOKU), Peter-Jordan Straße 82, Vienna 1190, Austria; 4 Environment Agency Austria, Spittelauer Lände 5, Vienna 1090, Austria; 5 Center for Health and Bioresources, Austrian Institute of Technology GmbH (AIT), Konrad-Lorenz-Straße 24, Tulln 3430, Austria; 6 Symbiocyte, Konrad-Lorenz-Straße 24, Tulln 3430, Austria; 7 Department of Forest Protection and Wildlife Management, Faculty of Forestry and Wood Technology, Mendel University in Brno, Zemědělská 3, Brno 613 00, Czech Republic

**Keywords:** carbon cycle, *Fagus sylvatica* (beech) forest, Gadgil effect, nitrogen mining, plant–soil feedback, priming, soil fungal communities, tree girdling

## Abstract

- Ectomycorrhizal (ECM) fungi can both accelerate and decelerate decomposition of organic matter in forest soils, but a mechanistic understanding of this differential influence is limited.
- Here, we tested how ECM fungi affect decomposition along a natural fertility gradient in a temperate forest of European beech. Trees were girdled to reduce belowground carbon supply to the soil.
- Girdling shifted soil fungal community composition and decreased hyphal biomass production and soil CO_2_ efflux, indicating a reduced ECM fungal activity. Girdling also affected decomposition processes, but the effects depended on fertility. Our results indicate that ECM fungi decelerate decomposition under conditions of low fertility while under conditions of high fertility ECM fungi and their host roots have an accelerating effect.
- We conclude that both acceleration and deceleration of decomposition of organic matter by ECM fungi can occur within a forest, with soil fertility determining the direction and magnitude of these effects. We suggest a positive feedback between fertility, stand productivity and soil carbon and nitrogen dynamics that is mediated to a large extent by ECM fungi.

## Introduction

Ectomycorrhizal (ECM) fungi play an important role in the carbon (C) and nitrogen (N) cycles of temperate and boreal forests. Living in symbiosis with trees, ECM fungi receive photosynthetically assimilated C in exchange for N and other soil nutrients (Read & Perez-Moreno, 2003; Van Der Heijden *et al*., 2015). By forming extraradical mycelia, ECM fungi can improve plant uptake of growth-limiting resources (Olsson *et al*., 2002). Increasingly recognized, but still poorly understood is the role of ECM fungi in the breakdown and recycling of litter and soil organic matter (SOM; Frey, 2019; Zak *et al*., 2019), one of the largest reservoirs for C and N in temperate and boreal forests.

Several ways in which ECM fungi mediate the decomposition of litter and SOM and associated C and N dynamics have been proposed. Ectomycorrhizal fungi are assumed to directly ‘mine’ organic substrates for N by enzymatic decay (Frey, 2019; Zak *et al*., 2019), when mineral nutrients are becoming increasingly scarce (Lang *et al*., 2016) and when ecosystems shift from a mineral to an organic nutrient economy (Phillips *et al*., 2013). Despite their soil N mining capability, most ECM fungi have, however, only a reduced genetic capacity to utilize soil C as compared to their saprotrophic fungal ancestors (Kohler *et al*., 2015; Pellitier & Zak, 2018). Indeed, there is a wide consensus that most ECM fungi do not fully metabolize C (Baldrian, 2009; Lindahl & Tunlid, 2015; Zak *et al*., 2019). Nitrogen mining by ECM fungi thus appears to be accompanied by an increase in the C : N ratio of litter and SOM (Orwin *et al*., 2011; Clemmensen *et al*., 2013; Smith & Wan, 2019). In turn, free-living saprotrophic microbes (both fungal and prokaryotic) competing for the same resources may become increasingly N-limited with concurrent N mining by ECM fungi, which reduces their ability to decompose litter and SOM (Fernandez & Kennedy, 2016). The phenomenon of a suppressed saprotrophic activity by ECM fungi is referred to as *Gadgil effect* and is suggested to decelerate decomposition and to reduce associated soil C losses (Gadgil & Gadgil, 1971; Fernandez & Kennedy, 2016). However, certain ECM fungal groups found in conditions of lower soil C stocks are suggested to be actively decomposing C while they mine SOM for N, or they at least complement free-living saprotrophs in their decomposition activity (Lindahl *et al*., 2021; Argiroff *et al*., 2022). Alternatively, ECM fungi have also been argued to stimulate free-living saprotrophs through C subsidies (e.g. exudates and necromass) to the rhizosphere for nutrient acquisition (Fernandez & Kennedy, 2016; Frey, 2019; Zak *et al*., 2019). This mechanism is known as a *priming effect* and has been demonstrated to accelerate decomposition and to increase associated C losses (Huo *et al*., 2017; Guenet *et al*., 2018; Bastida *et al*., 2019).

Recent field-based studies along environmental gradients, from local to continental scales, revealed strong interrelations among ECM fungal community composition, forest productivity, site fertility and soil C storage (Kyaschenko *et al*., 2017; Lindahl *et al*., 2021; Mayer *et al*., 2021; Anthony *et al*., 2022; Argiroff *et al*., 2022). However, despite indicative correlations between variables, none of the above studies put supposed mechanistic links of ECM-mediated decomposition to experimental tests. On the contrary, most site-specific manipulation experiments investigating the role of ECM fungi in C and N cycles (e.g. by tree girdling or root trenching) did not directly link their results to the prevalent forest productivity, fertility, or soil C storage (Gadgil & Gadgil, 1975; Brzostek *et al*., 2015; Averill & Hawkes, 2016; Sterkenburg *et al*., 2018; Lang *et al*., 2021). These shortcomings limit our current understanding of whether, or rather where and when, ECM fungi are shaping forest ecosystem properties/functioning (e.g. soil C storage) through their impact on litter and SOM decomposition. Therefore, Fernandez and Kennedy (2016) previously suggested to apply manipulation treatments along natural ecosystem gradients to better understand the direction and magnitude of ECM fungal effects on decomposition.

Here, we established a tree girdling experiment along a natural fertility gradient in a temperate mountain forest of European beech. Along the fertility gradient, litter and SOM decomposition was found to increase with increasing fertility (i.e. increasing nutrient availability) and stand productivity, and coincided with decreasing C contents and C : N ratios in the mineral topsoil (Mayer *et al*., 2021; Table 1). Tree girdling was conducted to disrupt the allocation of recent assimilates to ECM fungi to reduce their growth and activity (Högberg *et al*., 2001). This experimental set-up allowed us to test the following hypotheses on the influence of ECM fungi on decomposition and its dependence on soil fertility and associated soil C and N properties:

(1) ECM fungi decelerate litter and SOM decomposition (*deceleration hypothesis*). As girdling is predicted to reduce ECM fungal competition with free-living saprotrophs, decomposition will increase following girdling.
(2) ECM fungi accelerate litter and SOM decomposition by active decomposition and/or stimulation of free-living saprotrophs (*acceleration hypothesis*). Accordingly, decomposition will decrease following girdling and an associated reduction in ECM fungal activity.
(3) Soil fertility determines the magnitude of the ECM fungal effects on decomposition. The decelerating effect of ECM fungi on decomposition will be stronger at the low fertile end of the gradient, where trees are expected to be more dependent on N supply from their fungal symbionts. In contrast, the accelerating effect of ECM fungi on decomposition will be stronger at the fertile end of the gradient, contributing to the faster decomposition of litter and SOM and the lower soil C contents observed there (*fertility hypothesis*).

To test these *a priori* hypotheses, we combined field and laboratory measurements of soil CO_2_ efflux and litter bag experiments to assess the response of litter and SOM decomposition and associated C losses before and after girdling. In addition, we analysed hyphal biomass production, soil fungal communities and different N forms in soils to assess the interactions between fertility, productivity, fungal lifestyle groups/genera, ECM exploration types and soil C and N dynamics.

## Materials and Methods

### Site description and tree girdling

The study site was in the Reichraminger Hintergebirge, a mountain range in the Austrian Calcareous Alps (47°49^'^08^''^N, 14°23^'^34^''^E). The forest stand is dominated by European beech (*Fagus sylvatica* L.) with a stand age of *c*. 146 yr in 2015. The site is southeast exposed with a slope inclination of 35° at an elevation of 1000–1100 m above sea level (Supporting Information Fig. S1). The parent bedrock is limestone. Average annual air temperature and precipitation are 7.8°C and 1645 mm, respectively (Kobler *et al*., 2015). Tree regeneration is mostly absent and ground vegetation sparse. Detailed site information can be found elsewhere (Gorfer *et al*., 2021; Mayer *et al*., 2021).

At the site, a natural fertility gradient along the contour line exists, with soil types ranging from shallow Rendzic Leptosols at low fertility conditions to loamy Chromic Cambisols at high fertility conditions (Fig. S1; Mayer *et al*., 2021). Three experimental blocks were selected along the fertility gradient (Fig. S1) characterized by different stand and soil properties (Table 1) and referred to as ‘low’, ‘medium’ and ‘high’ fertility levels in the following. In brief, standing tree biomass and total soil inorganic nutrient stocks increased, while topsoil organic C content as well as C : N ratios decreased along with increasing fertility levels. Two high fertility plots, two low fertility plots and four medium fertility plots were established, all sized 25 9 25 m. The medium fertility plots served also for another experiment, explaining the greater number of plots. Within each plot, four subplots were randomly selected. After establishing the experimental site in May 2015, belowground C allocation was severed by girdling all trees in half of the plots (‘girdling’ plots) in August 2015 (Fig. S1). For that, a 15 cm strip of bark and cambium around the stems (at breast height) was cut off using handsaws and chisels. The halves without girdling served as undisturbed ‘control’ plots; edge effects on subplots in control and girdling plots were minimized by keeping > 10 m to girdled or nongirdled trees, respectively. Soil parameters of control and (later) girdled plots were determined pregirdling to ensure their comparability (Table S1).

### Soil CO_2_ efflux and soil microclimate

From May 2015 to November 2016 (pre- and postgirdling), soil CO_2_ efflux was measured monthly at each plot (during the snow-free period) with a mobile infrared gas analyser (EGM-4; PP Systems International Inc., Amesbury, MA, USA). Measurements were conducted by placing a respiration chamber (SRC-1; PP Systems International Inc.) on preinstalled collars (*n* per plot = 4, insertion depth = 3 cm). At the same time, soil temperature (5 cm depth) and soil moisture (0–7 cm) were measured in proximity to the collars using a hand-held thermometer and a TDR sensor (Field Scout TDR Soil Moisture Meter; Spectrum Technologies Inc., Plainfield, IL, USA). To avoid a temporal sampling bias, the measurement order was randomized between dates.

### Litter decomposition

Decomposition in the organic layer was assessed for the postgirdling period using litter bags. For this, beech litter was collected via litter traps from early October to mid-November 2015. Collected foliage litter was dried (50°C, 48 h). A subsample was dried at 105°C for weight conversion factors. Litter bags made from polyethylene nets (1 mm mesh size, *c*. 10 9 10 cm, double-layered) were filled with 3 g of dried beech litter (only litter dried at 50°C was used). In November 2015, litter bags were installed in the litter layer using high-carbon steel pins. At each plot, two litter bags were installed at four locations, which were collected carefully and in equal shares in May and November 2016. After drying (40°C), the bags were cut-opened. Ingrown roots and mineral soil particles washed into the mesh bags were carefully removed. Thereafter, litter was dried (105°C, 48 h) and weighed for each bag. Litter C concentrations were determined for the initial litter and for litter collected in May and November. Litter was pooled per plot and date, ground (Pulverisette 5; Fritsch GmbH, Idar-Oberstein, Germany) and subsequently analysed for C using a TruSpec CHN analyser (Leco, St Joseph, MI, USA). Mass of litter C was calculated for each bag by multiplying C concentration with litter dry weight. Litter decomposition was determined as relative mass loss of litter C.

As a proxy for potential decomposition in mineral soils, two types of tea bags were used as standardized substrates: green tea as surrogate for easily decomposable substrates and red tea as surrogate for slowly decomposable substrates (Keuskamp *et al*., 2013). Tea bags were incubated in the vicinity of collars for soil CO_2_ efflux measurements from May to August 2015 (pregirdling) and from May to August 2016 (postgirdling), respectively. Insertion depth was 5 cm from the mineral soil surface. In August, bags were cleaned, dried (105°C, 48 h) and weighed to determine mass loss (Keuskamp *et al*., 2013). We are aware that with the tea-bag method we analyse a proxy for SOM decomposition in mineral soils rather than an explicit measure of this process. However, the method has been successfully used to compare decomposition potentials across sites, ecosystems and biomes based on a standardized protocol (Djukic *et al*., 2018; Trevathan-Tackett *et al*., 2021). In-depth analyses of tea chemistry further suggest that changes in C compounds in both tea types during decomposition are comparable to other litter studies (Duddigan *et al*., 2020).

### Hyphal biomass production by ECM fungi

In October 2015, two ingrowth bags were installed in proximity to each CO_2_ efflux collar to determine ECM hyphal biomass production (Wallander *et al*., 2001). In total, 64 bags filled with 13 g of quartz sand were incubated at 5 cm mineral soil depth. Within each plot, two additional bags were installed within root/ECM-exclusion tubes (10 cm diameter, 30 cm insertion depth) to estimate ingrowth by saprotrophic fungi (Mayer *et al*., 2017b). Hyphal ingrowth was estimated under a stereo microscope (910–40). The hyphal biomass was ranked in five classes according to Wallander *et al*. (2004): no hyphae (0); occasional hyphae (1); sparse mycelia (2); mycelia present but no aggregation of sand particles (3); and plenty of mycelia and some aggregation of the sand particles (4). Hyphal ingrowth was averaged for the two bags per collar for further analysis (*n*= 4–8). Hyphal ingrowth into bags installed in root/ECM-exclusion tubes was subtracted from ‘regular’ ECM ingrowth bags to account for saprotrophic fungal ingrowth.

### Soil sampling and laboratory analyses

In May and August 2015 (pregirdling), and October 2015 and May, August and October 2016 (postgirdling), samples were taken from the mineral topsoil (0–10 cm, *c*. 1000 cm^3^ soil volume) of each subplot using a shovel after carefully removing the organic layer. We focused on the mineral topsoil, as the organic layer was rather shallow and the mineral topsoil layer had the highest C contents compared with other horizons (Table 1; see also Mayer *et al*., 2021). In May 2015, three out of four sub-plots per plot were sampled, while at other dates, all subplots were sampled (total *n* = 152). Soil samples were immediately sieved (2 mm) in the field. For fungal community analysis, 0.5 g of homogenized mineral soil was weighed into 1.5 ml LifeGuard Soil Preservation Solution (Mo Bio, Carlsbad, CA, USA). Mineral soil for other analyses was stored at 4°C until further processing.

Total C content (%) of all soil samples was analysed based on a 300 mg subsample using a TruSpec CHN analyser (Leco). Sub-samples were dried (105°C, 24 h) and ground before analysis (as above). Inorganic C content was determined by the Scheibler method (ÖNORM L 1084, 1999); organic C content was calculated as the difference in inorganic and total C concentration.

Microbial respiration was measured *in vitro* on all soil samples (equivalent to *c*. 25 g oven-dried soil) within a few days after sampling. In brief, soil was sieved (< 2 mm), cleaned from roots and filled in 200 cm^3^ steel cylinders at field bulk density (Reichstein *et al*., 2000; Schindlbacher *et al*., 2015). Cylinders were equilibrated at 4°C for *c*. 3 d. Subsequently, cylinders were placed into 2-l plastic containers that were connected to an infrared gas analyzer (SBA-4; PP Systems International Inc.). Microbial respiration rates of each sample were determined at 15°C as ΔCO_2_ within closed containers for Δ6 min. Respiration rates were expressed in μg C g^−1^C d^−1^. Details on the measurement system and protocol can be found elsewhere (Mayer *et al*., 2017a,b). We wish to draw to attention that *in vitro* respiration assays may have several shortcomings (e.g. loss of ECM root tips during sieving, soil aggregate disruption) and may not reflect *in situ* decomposition rates and associated C losses. However, a study comparing intact (incl. roots) and sieved soil cores suggested only marginal effects on microbial respiration rates due to soil homogenization by sieving (Schindlbacher *et al*., 2015). Also, in an earlier study at the same site, we found a robust correlation between microbial respiration and the activity of enzymes involved in the breakdown of SOM (Mayer *et al*., 2021), suggesting that microbial respiration is a reasonable proxy for SOM decomposition in the studied mineral soils.

Extractable N concentration was determined for all soil samples. For that, 5 g fresh soil was shaken in 25 ml of 0.5 M K_2_SO_4_ for 1 h, then centrifuged and filtered (Whatman cellulose acetate filter; Cytiva, Marlborough, MA, USA). Soil extracts were analysed for total dissolved N with a Shimadzu TOC-L (Shimadzu Corp., Kyoto, Japan). Soil nitrate and soil ammonium concentrations were measured on samples retrieved in August 2016. In brief, 5 g of fresh soil was shaken in 50 ml of 1 M KCl for 2 h. Soil extracts were subsequently filtered (as above), and nitrate was determined photometrically (540 nm) using vanadium(III) as reductant (Miranda *et al*., 2001). Ammonium was determined photometrically (660 nm) using the indophenol blue method (Rhine *et al*., 1998). Nitrogen forms are expressed as μg or mgN g^−1^ C.

### Soil fungal community analysis

Soil DNA isolation and fungal community analyses were done as described in Gorfer *et al*. (2021). In brief, DNA was extracted from the soil samples stored in LifeGuard Soil Preservation at 4°C within a week after sampling using the PowerSoil-htp 96 Well Soil DNA Isolation Kit (Mo Bio). The fungal ITS2-region was amplified with primer pair ITS3Mix_NeXTf and ITS4Mix_ NeXTr (modified from Tedersoo *et al*., 2015). Illumina MiSeq sequencing was carried out at the NGS Unit of the Vienna Biocenter Core Facility GmbH (Vienna, Austria). Raw sequence reads were quality filtered with Trimmomatic v.0.36 (Bolger *et al*., 2014). USEARCH scripts v.9.0.2132 (Edgar, 2010) were used for merging paired-end reads, chimaera detection and filtering of underrepresented sequences (< 10). OTUs were clustered at 97% sequence identity with VSEARCH (Rognes *et al*., 2016). Nonfungal sequences were excluded from further analyses, and the final dataset was rarefied to 3058 reads per sample. For community analysis, fungal OTUs were taxonomically grouped at genus or closest taxonomic level. Fungal genera were assigned to the following primary lifestyle groups/guilds (Gorfer *et al*., 2021): ectomycorrhizal fungi, other symbiotic fungi (e.g. species with unspecific mycorrhizal lifestyle or arbuscular mycorrhizas), saprotrophic ascomycetes and basidiomycetes, other saprotrophic fungi (e.g. *Mortierella*, Rhizophydiales and *Mocur*), plant pathogenic fungi and those of unknown lifestyle. Ectomycorrhizal fungi were further grouped according to their exploration type (Agerer, 2006). Due to a low relative abundance of medium-distance exploration types (< 1%), they were merged with short-distance exploration types. Ratios between relative abundances of ECM fungi and all saprotrophic fungi were calculated for control plots.

Total fungal DNA in mineral soil was quantified by qPCR with FungiQuant primers (Liu *et al*., 2012) following the protocol of Unterwurzacher *et al*. (2018) with a modified assay volume of 10 ll. The qPCR standard was prepared by mixing equal amounts of genomic DNAs from *Penicillium canescens* NG_p02, *Trichoderma harzianum* NG_p29 and *Tritirachium* sp. gab0401. Only samples from the first study year were analysed.

### Statistical analysis

Statistical analysis and plotting were conducted in R (R Core Team, 2021). Linear mixed effects models and ANOVA functions of the NLME R package (Pinheiro *et al*., 2014) were used to test for the fixed effects of girdling, fertility and sampling date on response variables. Data were tested for normal distribution and variance homogeneity before analysis. To meet the requirements for ANOVA, a variable variance structure term was incorporated in the model and data were *log*-transformed (Zuur *et al*., 2009). Models were applied to data collected pre- and postgirdling, and separately for fertility levels, respectively. Linear mixed effects models were also used to test for correlations between microbial respiration rates, N forms and relative abundances of ECM fungi, most abundant ECM genera and ECM exploration types. Sub-plots within plots were considered as random effects in all models to account for a repeated measurement structure. Nonmetric multidimensional scaling (NMDS; VEGAN R package (Oksanen *et al*., 2016)) was used to analyse the community composition of ECM and saprotrophic fungi in control and girdling plots for the postgirdling period, based on the relative abundance of 288 taxonomic groups. Level of significance for all statistical analyses was set at *P* < 0.05.

## Results

### Carbon fluxes

**Fig. 1:**
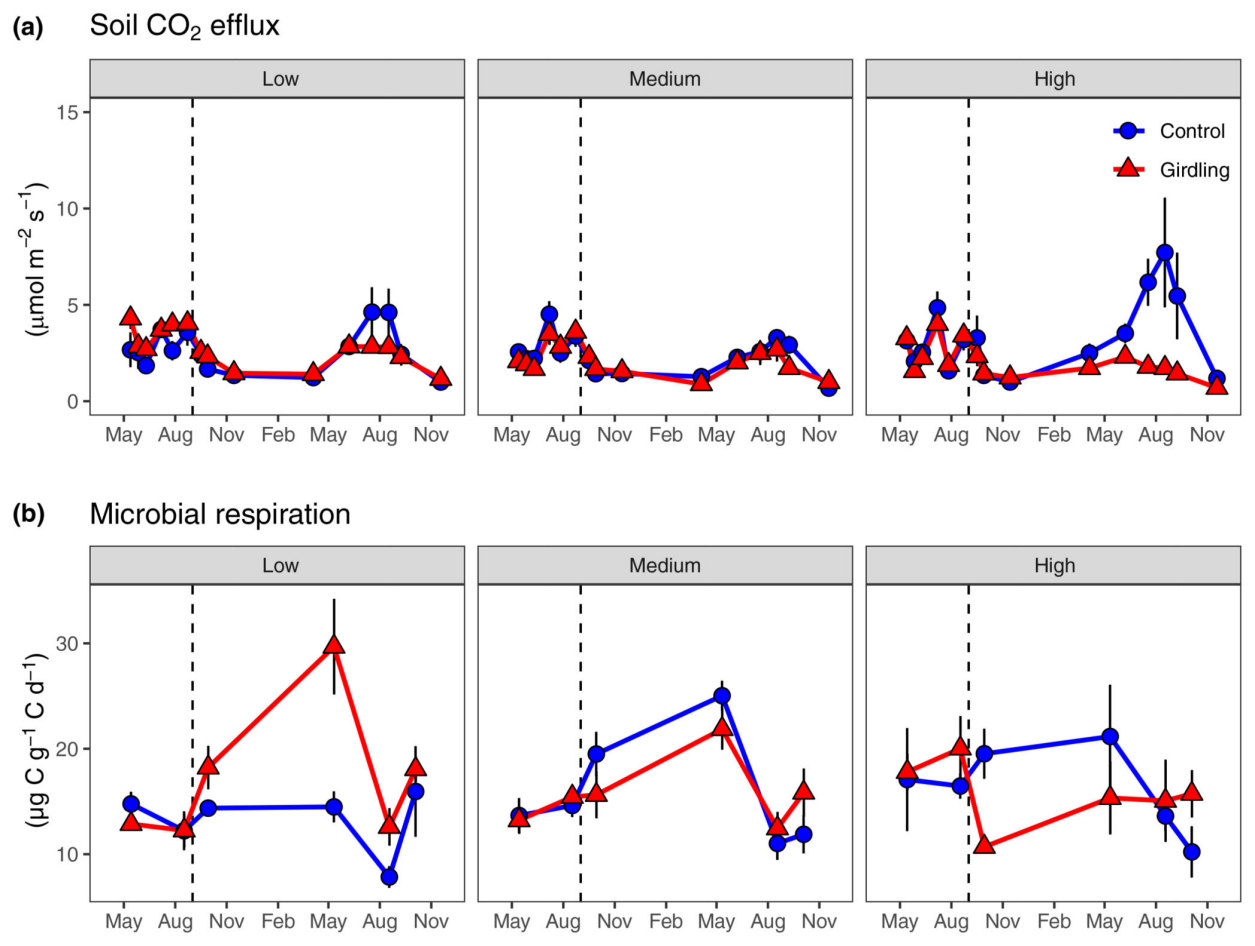
*In situ* soil CO_2_ efflux (a) and *in vitro* microbial respiration rates from mineral soil (0–10 cm) (b) at three fertility levels (low, medium and high) and as affected by tree girdling in a temperate *Fagus sylvatica* forest (mean ± SE; *n* = 4–8). Dashed vertical lines indicate date of girdling during the study period May 2015–November 2016. Test statistics are given in Table 2.

In the pregirdling period, control and subsequently girdled plots did not differ regarding soil CO_2_ efflux and *in vitro* microbial respiration rates (Table S1). While soil CO_2_ efflux was not significantly affected by fertility, microbial respiration rates increased significantly with increasing fertility level (Fig. 1; Tables 2, S1). After girdling, soil CO_2_ efflux decreased significantly (Fig. 1a; Table 2), with the girdling effect increasing with increasing fertility level (*P*_Girdling × Fertility_ = 0.033). Girdling decreased soil CO_2_ efflux by on average 9 and 55% at low and high fertility level plots, respectively. The effect of girdling on soil CO_2_ efflux was not significant at low fertility, but at medium and high fertility levels (Table S2). Regarding microbial respiration rates, girdling effects depended on fertility levels (*P*_Girdling × Fertility_ = 0.039, Table 2): At low fertility, respiration rates increased by 49% after girdling, while at medium and high fertility, they decreased by 2 and 12%, respectively. On low fertility plots, the girdling effect on microbial respiration was significant, at medium fertility plots, it was not significant, and at high fertility plots, it was only significant during the first 10 months after girdling as indicated by a significant interaction with sampling date (Fig. 1b; Table S2).

### Substrate decomposition

**Fig. 2:**
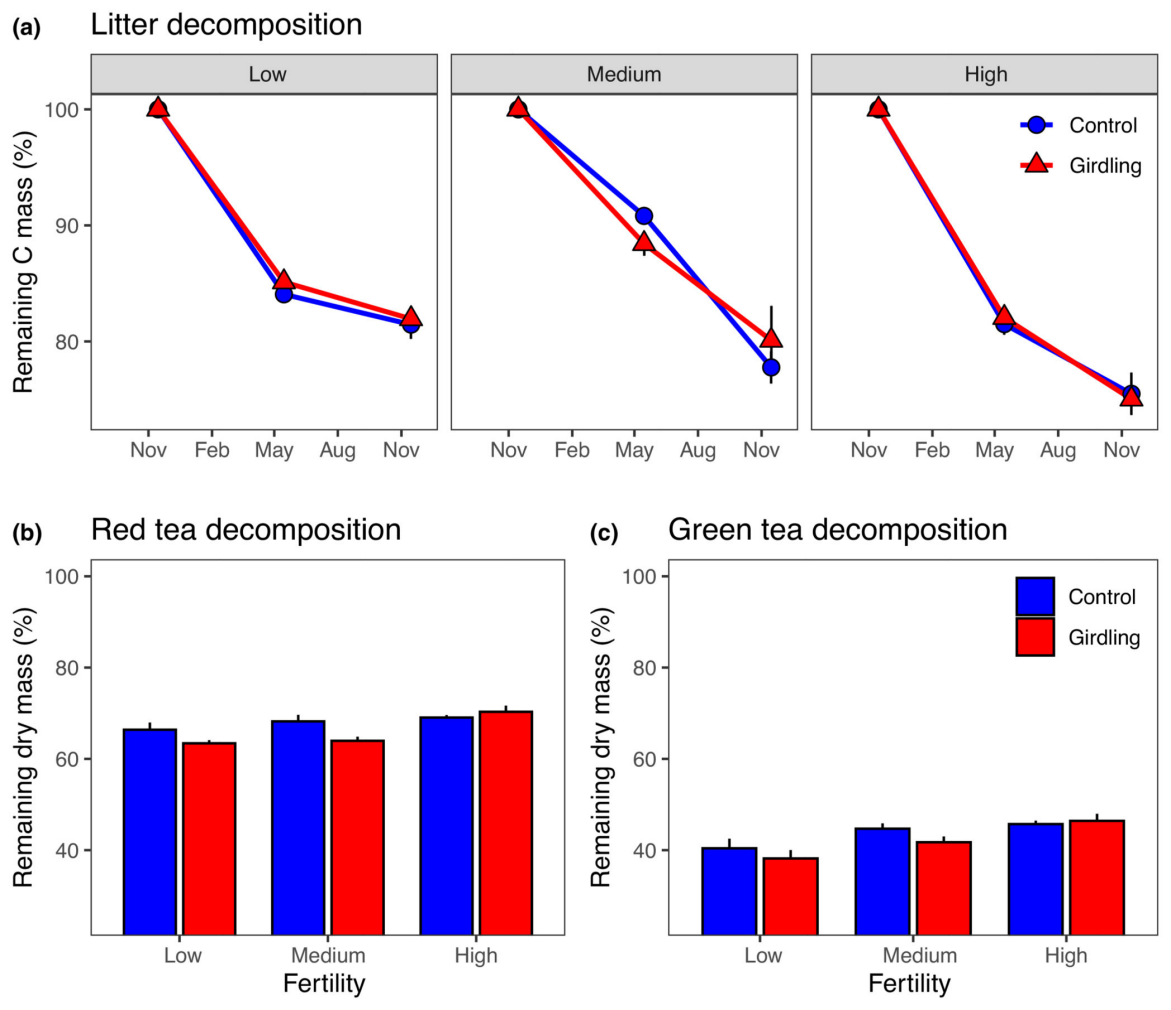
Decomposition of beech litter (a), red tea (b) and green tea (c) at three fertility levels (low, medium and high) and as affected by tree girdling in a temperate *Fagus sylvatica* forest (mean ± SE; *n* = 3–8). Decomposition is displayed as remaining carbon (C) mass (%) for litter and remaining dry mass (%) for tea bags, respectively. The postgirdling period November 2015–November 2016 is given in (a). Test statistics are given in Table 2.

Girdling had no effect on the decomposition of beech litter C assessed by litter bags (Fig. 2a; Table 2). However, litter C mass loss increased significantly with increasing fertility level, ranging from 18% at low fertility to 25% at high fertility plots (*P*_Fertility_ = 0.006). In the pregirdling period, control and subsequently girdled plots did not differ regarding decomposition of red and green tea in mineral soil (Table S1). However, following girdling, the decomposition of red tea increased by *c*. 9% at low and medium fertility levels as compared to the control, but not at high fertility (Fig. 2b; Table 2). Girdling had no overall significant effect on the decomposition of green tea, but the mass loss response to girdling followed a similar pattern as for red tea (Fig. 2c; Table 2).

### Nitrogen forms and soil microclimate

**Fig. 3:**
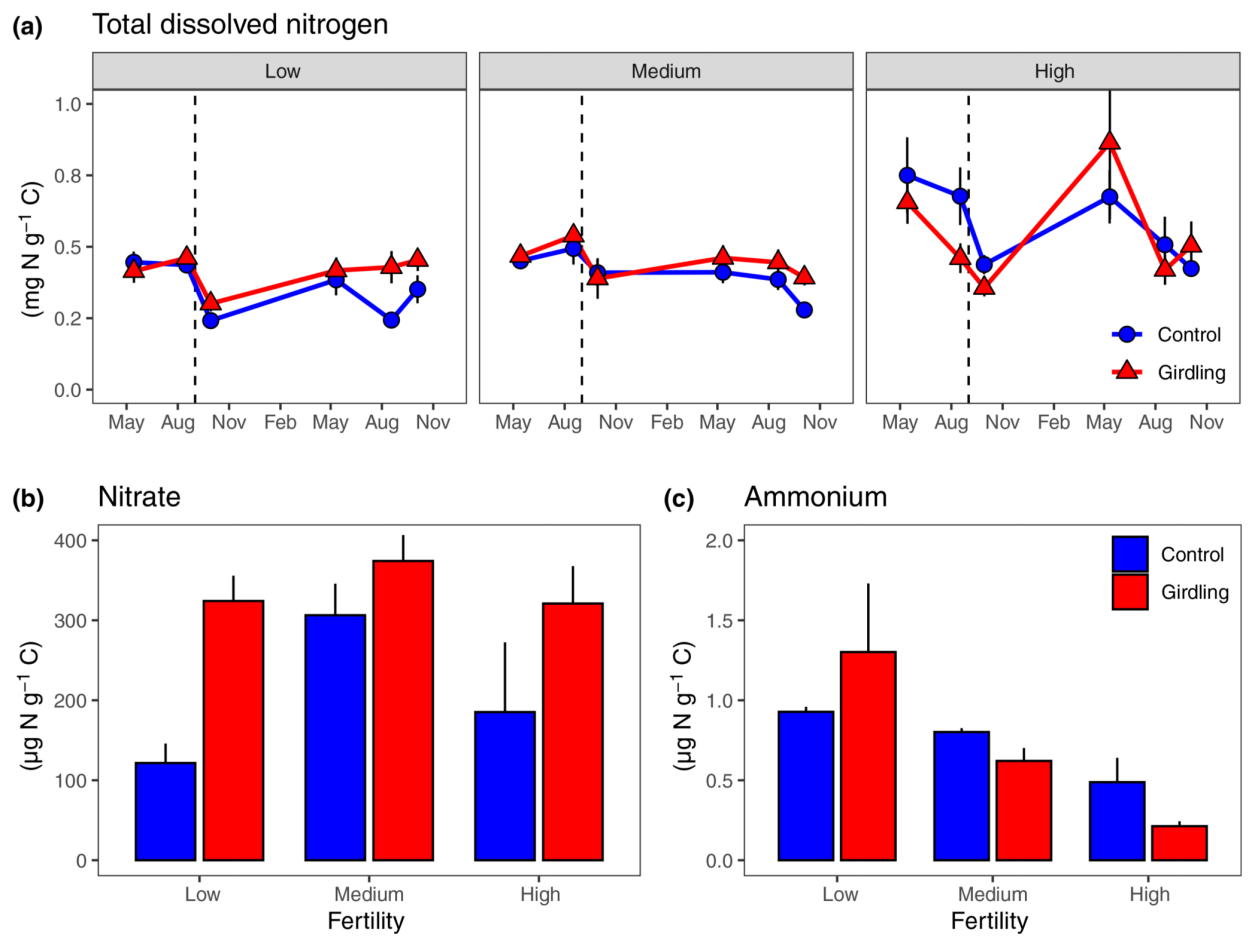
Total dissolved nitrogen (a), nitrate (b) and ammonium (c) concentrations in the mineral topsoil (0–10 cm) at three fertility levels (low, medium and high) and as affected by tree girdling in a temperate *Fagus sylvatica* forest (mean ± SE; *n* = 4–8). Dashed vertical lines in A indicate date of girdling during the study period May 2015–November 2016. Test statistics are given in Table 2. Note: nitrate and ammonium were measured in August 2016 only.

In the pregirdling period, control and subsequently girdled plots showed the same concentration of total dissolved nitrogen in mineral soil (Fig. 3a; Table S1). After girdling, total dissolved N and nitrate concentrations were significantly higher in girdled plots (Fig. 3a,b; Table 2). In contrast, girdling had a negative effect on ammonium concentrations in the mineral soil of medium and high fertility plots (Fig. 3c; Table 2). On average, total dissolved N and nitrate concentration increased significantly with increasing fertility level, while ammonium concentrations decreased.

Soil temperature and moisture did not differ between control and girdled plots throughout the study (Fig. S2; Tables 2, S1). Soil moisture was significantly greater at the higher fertility levels (Fig. S2; Table 2).

### Hyphal biomass production, fungal DNA, relative fungal abundances and community composition

**Fig. 4:**
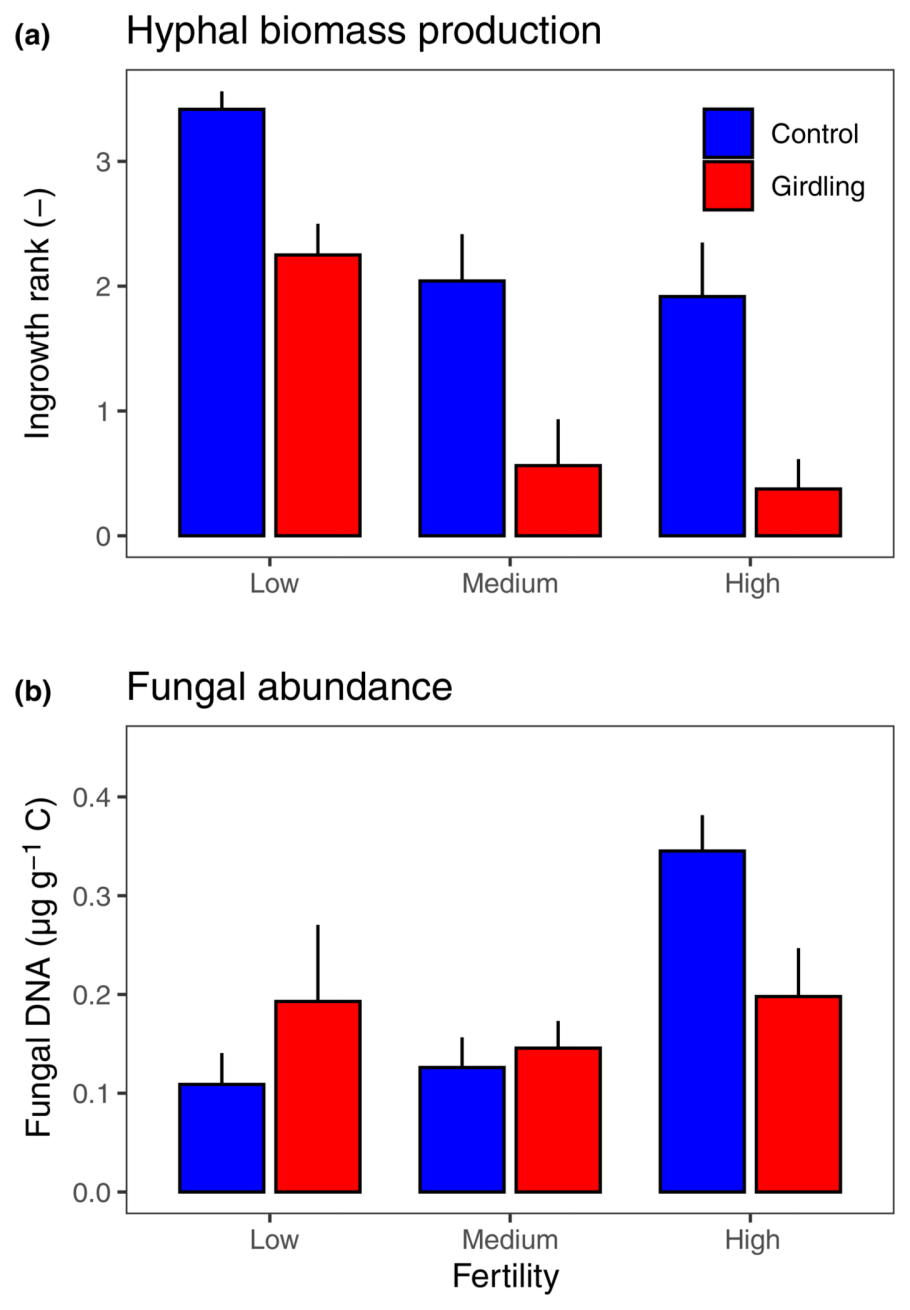
Ectomycorrhizal hyphal biomass production (a) and total amount of fungal DNA (b) in mineral soils at three fertility levels (low, medium and high) and as affected by tree girdling in a temperate *Fagus sylvatica* forest. Fungal DNA was measured at 0–10 cm depth and hyphal biomass production was assessed by ranking ingrowth of biomass into five classes (0–4) in ingrowth bags incubated at 5 cm depth (mean ± SE; *n* = 4–8). Test statistics are given in Table 2.

Hyphal biomass production of ECM fungi as assessed by ingrowth bags was significantly related to fertility, with the greatest and lowest hyphal biomass production rates at low and high fertility levels, respectively. After *c*. 1 yr of girdling, hyphal biomass production was significantly reduced across all fertility levels (Fig. 4a; Table 2). The total amount of fungal DNA was similar among control and girdling plots before treatment establishment but increased significantly with increasing fertility level (Table S1). Fungal DNA was significantly affected by girdling, but the effect depended on fertility (*P*_Girdling × Fertility_ = 0.048); at low fertility, it increased in response to girdling, at medium fertility, it remained unchanged, while at high fertility, it decreased (Fig. 4b).

**Fig. 5:**
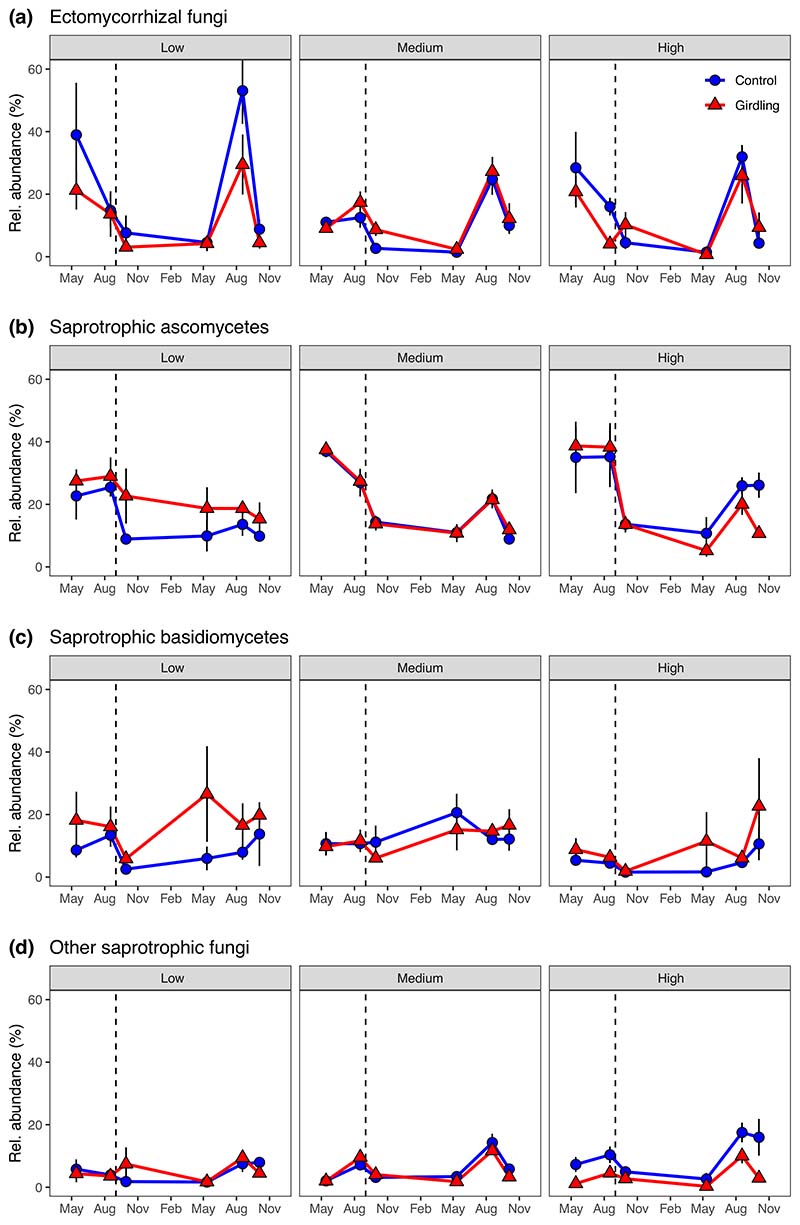
Relative abundance of ectomycorrhizal fungi (a), saprotrophic ascomycetes (b), saprotrophic basidiomycetes (c) and other saprotrophic fungi (e.g. moulds) (d) in mineral soils (0–10 cm) at three fertility levels (low, medium and high) and as affected by tree girdling in a temperate *Fagus sylvatica* forest (mean ± SE; *n* = 3–8). Dashed vertical lines indicate date of girdling during the study period May 2015–November 2016. Test statistics are given in Table 2.

**Fig. 6:**
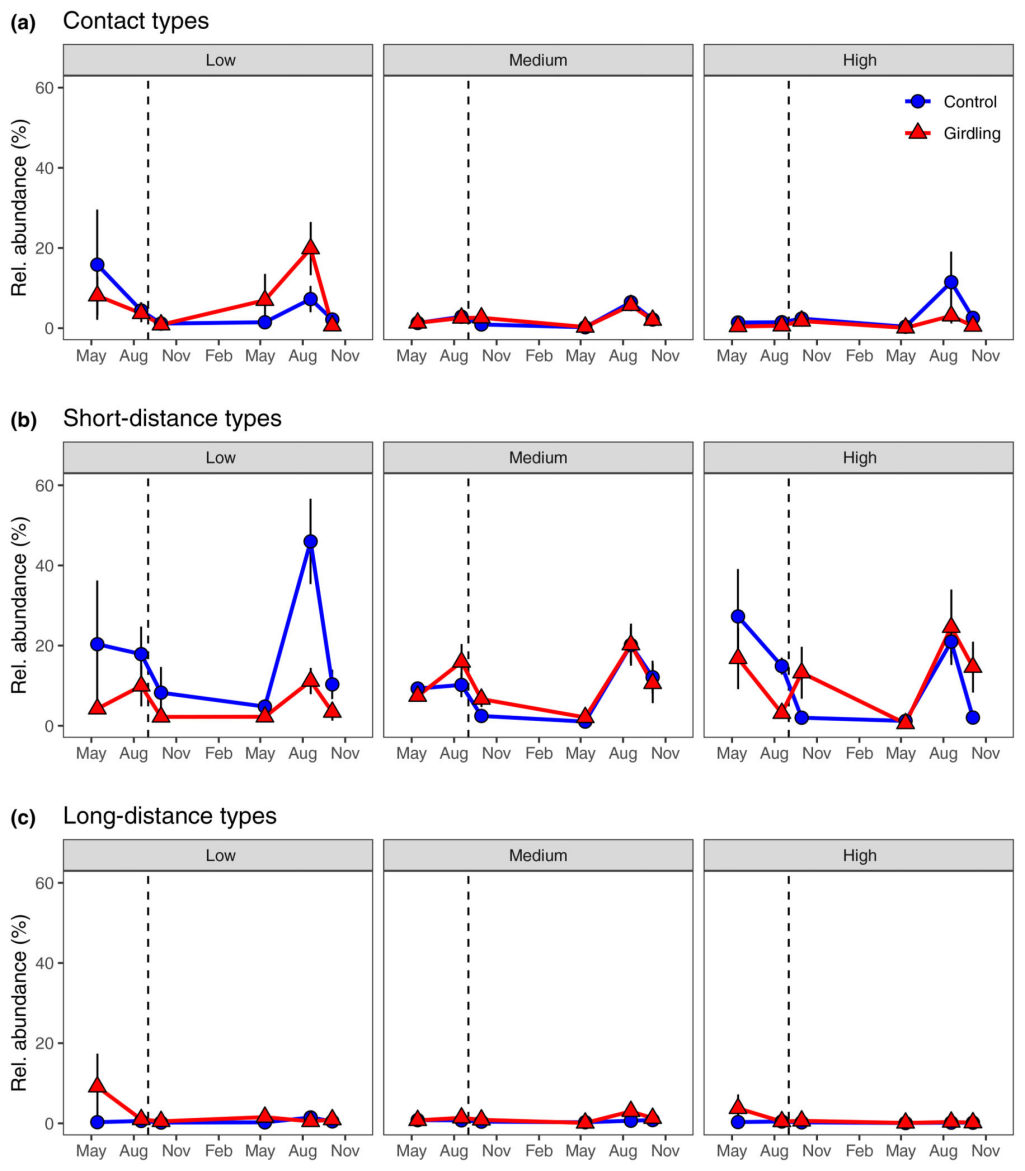
Relative abundance of ectomycorrhizal fungal exploration types, separated into contact types (a), short-distance (b) and long-distance (c) exploration types in mineral soils (0–10 cm) at three fertility levels (low, medium and high) and as affected by tree girdling in a temperate *Fagus sylvatica* forest (mean ± SE; *n*= 3–8). Dashed vertical lines indicate date of girdling during the study period May 2015–November 2016. Test statistics are given in Table 2.

Before girdling, there was no difference between control and subsequently girdled plots in relative abundances of ECM fungi, ECM exploration types and saprotrophic groups, as analysed by high-throughput sequencing of the ITS2-region (Figs 5, 6; Table S1). No significant girdling or fertility effect on relative abundances of total ECM fungi and long-distance exploration type ECM fungi was observed after severing belowground C allocation through girdling (Figs 5a, 6; Table 2). In contrast, girdling affected the relative abundance of contact and short-distance exploration type ECM fungi, but the effects depended on fertility (*P*_Girdling × Fertility_ < 0.05; Fig. 6; Table 2): At the high fertility level, relative abundance of contact types decreased after girdling, while at medium and low fertility, it remained unaffected; relative abundance of short-distance types decreased after girdling at low fertility, but remained stable at medium and high fertility (Table S2). Likewise, girdling effects on the relative abundance of saprotrophic ascomycetes depended on fertility (*P*_Girdling × Fertility_ = 0.004; Fig. 5b; Table 2): At the low fertility level, relative abundance of saprotrophic ascomycetes was greater, at medium fertility, it remained unaffected, and at high fertility, it decreased after girdling (Table S2). Relative abundances of saprotrophic basidiomycetes and of other saprotrophic fungi increased and decreased in response to girdling at low and high fertility plots, respectively (Fig. 5c,d; Table S2). Relative abundances of other symbiotic and plant pathogenic fungi were not affected by girdling (Fig. S3; Table 2).

**Fig. 7:**
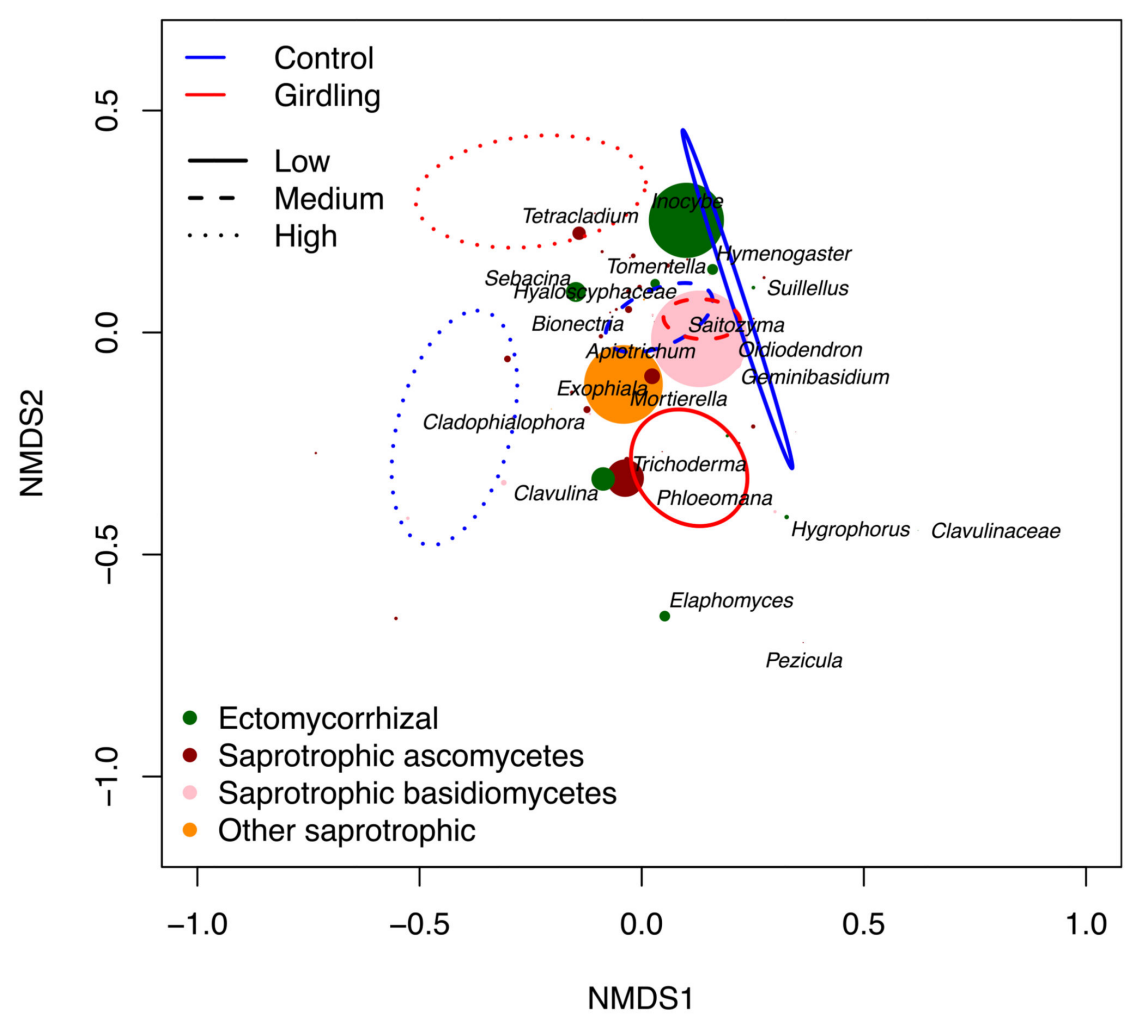
Nonmetric multidimensional scaling (NMDS) plot of the community composition of ectomycorrhizal and saprotrophic fungi in the mineral soil (0–10 cm) at three fertility levels (low, medium and high) and as affected by tree girdling in a temperate *Fagus sylvatica* forest. Ellipses represent ordination confidence intervals (95%) for control (blue) and girdling (red) plots at different fertility levels (indicated by different line types). Taxonomic grouping occurred at genus level or closest taxonomic level. Colour-coded fungal lifestyles were assigned; symbol size gives an indication of relative abundance. To increase readability, only most abundant groups are shown.

On the genus level, the ECM fungal community in mineral soil was dominated by *Inocybe*, *Clavulina* and *Sebacina*, and the saprotrophic community by *Mortierella*, *Apiotrichum*, *Trichoderma*, *Exophiala*, *Saitozyma* and *Tetracladium* (Fig. 7; Table S3). Plant pathogenic fungi were dominated by *Neonectria*, *Dactylonectria* and *Ilyonectria*, and other symbiotic fungi by the unspecific mycorrhizal group of *Sebacinaceae*(Table S3).

The NMDS analysis showed a clear separation of the ECM and saprotrophic fungal community among control and girdling plots along the fertility gradient (Fig. 7). Most striking is the dominance of the ECM fungi *Inocybe* at the low fertility control plot, while the relative abundance of this genus strongly decreased at the corresponding girdling plot.

### Correlations between microbial respiration, nitrogen and fungi

Microbial respiration rates were positively correlated with total dissolved N and nitrate, but no correlation was found with ammonium concentrations (Table 3). Microbial respiration rates were also negatively correlated with the relative abundance of all ECM fungi and of contact and short-distance exploration types, as well as with the relative abundance of *Inocybe* spp., *Hymenogaster* spp. and *Tomentella* spp. (Table 3).

## Discussion

Tree girdling successfully severed belowground C allocation to ECM fungi. Soil CO_2_ efflux rates decreased significantly, reflecting a reduced autotrophic respiration from roots and ECM fungi after girdling (Högberg *et al*., 2001; Heinemeyer *et al*., 2012) despite substantial stores of carbohydrates in tree roots and stem (Barbaroux *et al*., 2003). In parallel, girdling decreased ECM hyphal biomass production across the fertility gradient. Girdling also impacted microbial respiration, and decomposition of tea bags in mineral topsoils, and changed the soil fungal community composition as well as amounts of fungal DNA. We found support for both the *deceleration hypothesis*(H1) and the *acceleration hypothesis*(H2). In line with the *fertility hypothesis*(H3), our results provide evidence that the magnitude of ECM fungal effects on decomposition depends on soil fertility. However, the effects of ECM fungi on decomposition were restricted to the mineral topsoil and were not observed in the organic layer.

Under low fertility, microbial respiration and decomposition rates of red tea increased significantly following girdling, indicating a released suppression of free-living saprotrophs in mineral topsoil by ECM fungi. These results suggest that ECM fungi slow soil C cycling under low fertility conditions in the non-girdled control, confirming a Gadgil effect (Orwin *et al*., 2011; Averill & Hawkes, 2016; Fernandez & Kennedy, 2016). In contrast, at high soil fertility, microbial respiration rates decreased following girdling, indicating reduced C losses from SOM decomposition. This suggests that ECM fungi and their host roots accelerate the soil C cycle by enhancing decomposition under fertile conditions in the nongirdled control. Further support for the contrasting effects of ECM fungi on SOM decomposition along with fertility is given by *in situ* soil CO_2_ efflux measurements: while under low fertility, efflux rates decreased only by 9% after girdling, they dropped by 55% at the high fertility level. As soil CO_2_ efflux rates did not differ before girdling, this indicates that under low soil fertility conditions, reduced autotrophic respiration from roots and ECM fungi was possibly partially offset by CO_2_ released from enhanced decomposition. In contrast, soil CO_2_ efflux decreased strongly under high fertility, as both autotrophic and CO_2_ by heterotrophic decomposition were likely reduced following girdling.

The contrasting response of our decomposition proxies to girdling links to differences in soil C and N characteristics along the fertility gradient. An increase in C loss from microbial respiration and an increase in the decomposition of red tea after girdling were present only under low fertility conditions and were associated with high mineral soil C contents and wider C : N ratios (Mayer *et al*., 2021). This may indicate that decelerated decomposition by ECM fungi is a result of selective N mining by ECM fungi and an associated N limitation of free-living saprotrophs. Accordingly, an increased N availability after girdling – due to lowered tree uptake and/or increased N mineralization (Kaiser *et al*., 2011; Brzostek *et al*., 2015) – may have reduced such limitation, leading to accelerated decomposition. This assumption would be supported by the positive correlation between microbial respiration rates and total dissolved nitrogen and nitrate. Under more fertile conditions, in contrast, a decrease in decomposition after girdling coincided with lower mineral soil C contents and narrower C : N ratios. This may signify direct ECM decomposition (Lindahl *et al*., 2021; Argiroff *et al*., 2022) and/or a priming effect at the nongirdled control (Huo *et al*., 2017; Guenet *et al*., 2018; Bastida *et al*., 2019). However, ECM fungi with a high complement of SOM degrading genes – particularly from the genera *Cortinarius* or *Russula*(Bödeker *et al*., 2014; Kohler *et al*., 2015) – were largely absent at the studied site (Gorfer *et al*., 2021). We also found a negative correlation between the relative abundance of ECM fungal taxa and microbial respiration rates (Table 3), strongly suggesting that most ECM fungi would suppress decomposition at the studied site. Therefore, rather than ECM-mediated decomposition being accelerated, it seems more plausible that free-living saprotrophs at high fertility were stimulated by C subsidies to soil. Rhizosphere priming increases with increasing tree productivity and greater aboveground biomass as large amounts of the assimilated C are allocated to roots, ECM fungi and rhizodeposition (Hobbie, 2006; Jones *et al*., 2009; Huo *et al*., 2017). Since standing tree and root biomass stocks were greater under conditions of high fertility (Table 1), it is reasonable to speculate that rhizosphere C inputs to soil were also higher at the more fertile end of the gradient, thereby accelerating a coupled C and N cycle.

Although ECM fungal effects on decomposition plausibly link to C and N dynamics at low and high fertility plots, it does not mean that the underlying mechanisms are mutually exclusive. We rather assume their magnitudes to vary with fertility level. It is likely that the contrasting ECM fungal effects on decomposition even compensate for each other at medium fertility plots, as indicated by a fairly neutral response of the here used decomposition proxies to girdling. A diminished girdling effect at high fertility plots after *c*. 10 months might also indicate that the influence of reduced priming on decomposition was finally outbalanced by a released suppression of free-living saprotrophs. Similarly, Fernandez *et al*. (2020) reported that initial indications for priming diminished after a comparable time following root trenching.

Our results further suggest that the role of ECM fungi in SOM decomposition relates to soil fungal characteristics. Along with increasing fertility, hyphal biomass production of ECM fungi and the ratio between the relative abundance of ECM and saprotrophic fungi (Fig. S4) decreased, whereas total amounts of fungal DNA increased. These findings may point towards a higher competition pressure of ECM fungi on saprotrophic fungi at low fertile plots, where a Gadgil effect was observed in mineral soil (Fernandez & Kennedy, 2016; Fernandez *et al*., 2020). In support of a released competition pressure on these saprotrophic groups, the relative abundances of saprotrophic ascomycetes and basidiomycetes increased in response to girdling – largely on the expense of short-distance ECM exploration types. It is also likely that saprotrophic fungi replaced this ECM group to some extent, as total fungal DNA increased after girdling. This would be consistent with the observed increase in decomposition at the low fertility plot. In contrast, under highly fertile conditions, larger amounts of total fungal DNA may indicate more favourable conditions for fungi compared with the low fertility end of the gradient – possibly related to the larger belowground C input to soil, as discussed above. Since fungal DNA and the relative abundance of saprotrophic ascomycetes and other saprotrophic fungi decreased after girdling, the hypothesized stimulation of free-living saprotrophs under highly fertile conditions seems plausible.

Recent studies indicate that not only ECM fungi in general, but also certain genera or even specific taxa of ECM fungi may have a major influence on soil C cycling and decomposition. In Swedish boreal forests, for example, the presence of *Cortinarius acutus* s.l. in particular was associated with lower soil C storage (Lindahl *et al*., 2021). In one of the early studies by Gadgil and Gadgil (1978), *Inocybe* spp. were assumed to suppress decomposition, and more recently, *Tomentella* species were related to a ‘Gadgil effect’ in *Pinus* stands (Fernandez *et al*., 2020). In our study, relative abundances of *Inocybe*, *Hymenogaster* and *Tomentella* spp. were negatively correlated with microbial respiration rates. These species are common root symbionts in European beech stands, where they are associated with greater C : N ratios as observed in the low fertility plots (Wubet *et al*., 2012; Nacke *et al*., 2016; Gorfer *et al*., 2021). Taxa from both *Inocybe* and *Tomentella* were shown to have considerable genetic capacities to enzymatically break down organic substrates (Courty *et al*., 2005; Bahram *et al*., 2018), making them potential resource competitors for free-living saprotrophs. Although these taxa are known as short-distance exploration types (Põlme *et al*., 2020), it is plausible that their absolute abundance decreased after girdling, as indicated by the reduced hyphal ingrowth into mesh bags. Recently, Jörgensen *et al*. (2022) showed – in contradiction to previous assumptions – that ECM genera thought to produce little extraradical mycelium colonized ingrowth bags extensively, while taxa commonly associated with vast mycelial production (e.g. long-distance exploration type) occurred only sparsely in ingrowth bags. The dominance of *Inocybe* species among ECM fungi and their strong decrease in relative abundance after girdling under conditions of low fertility therefore allows the speculation that this genus is a main player within the complex processes underlying a ‘Gadgil effect’ in the studied beech forest.

Finally, we found no support for neither the *acceleration hypothesis* nor the *deceleration hypothesis* in the organic layer as assessed by the litter bag experiment. However, this result is consistent with the findings of other studies in temperate *Quercus* forests (Brzostek *et al*., 2015; Fernandez *et al*., 2020). In contrast, ECM fungal suppression of litter decomposition occurred mainly in coniferous forest with low-quality litter (i.e. high C to N, or high lignin to N ratios; Gadgil & Gadgil, 1975; Sterkenburg *et al*., 2018; Smith & Wan, 2019; Fernandez *et al*., 2020). It has been argued that high litter quality may enhance saprotrophic C use efficiency and growth, reducing the competitive advantage of ECM fungi (Fernandez *et al*., 2020). Thus, saprotrophic decomposers might benefit from higher quality beech litter (C : N ratio *c*. 50), evading N limitations even when ECM are present (Smith & Wan, 2019). However, we also found no evidence of a stimulation of litter decomposition by ECM fungi, contrasting the findings of a recent root-trenching study in a northern hardwood forest (Lang *et al*., 2021). Since our results indicate that ECM fungi neither accelerated nor decelerated litter decomposition, higher litter C mass loss rates along with increasing fertility levels are likely related to other factors, such as moisture. Earthworms may also play a crucial role in litter decomposition at fertile plots with deeper, loamy subsoils (Barthod *et al*., 2020), a factor that was, however, excluded by the use of fine-meshed litter bags.

Our conclusions are constrained by the logistic restrictions of tree girdling, allowing only the establishment of a limited number of large-enough forest plots (25 9 25 m) along the fertility gradient. We have attempted to overcome the shortcoming of limited spatial replication with temporal pseudo-replications. Moreover, we conducted two sampling campaigns before girdling to ensure that soil variables did not differ between pregirdled and control plots, and that postgirdling differences in measured variables represented a real response to the treatment. In an earlier study at the site, including twice the number of plots (note: before girdling) we further confirmed that the changes in soil properties (e.g. soil C and N, microbial respiration) and fungal communities along the gradient clearly related to fertility and stand productivity, respectively (Mayer *et al*., 2021). We also want to mention that C flux measurements after girdling may be biased by increased decomposition of dead hyphae and fine roots, which is a common problem for this kind of manipulation treatment. Finally, it is important to note that girdling had no significant effect on soil temperature and moisture, making it unlikely that our results were biased by altered microclimatic conditions as observed after root trenching (Subke *et al*., 2006; Mayer *et al*., 2017b).

**Fig. 8:**
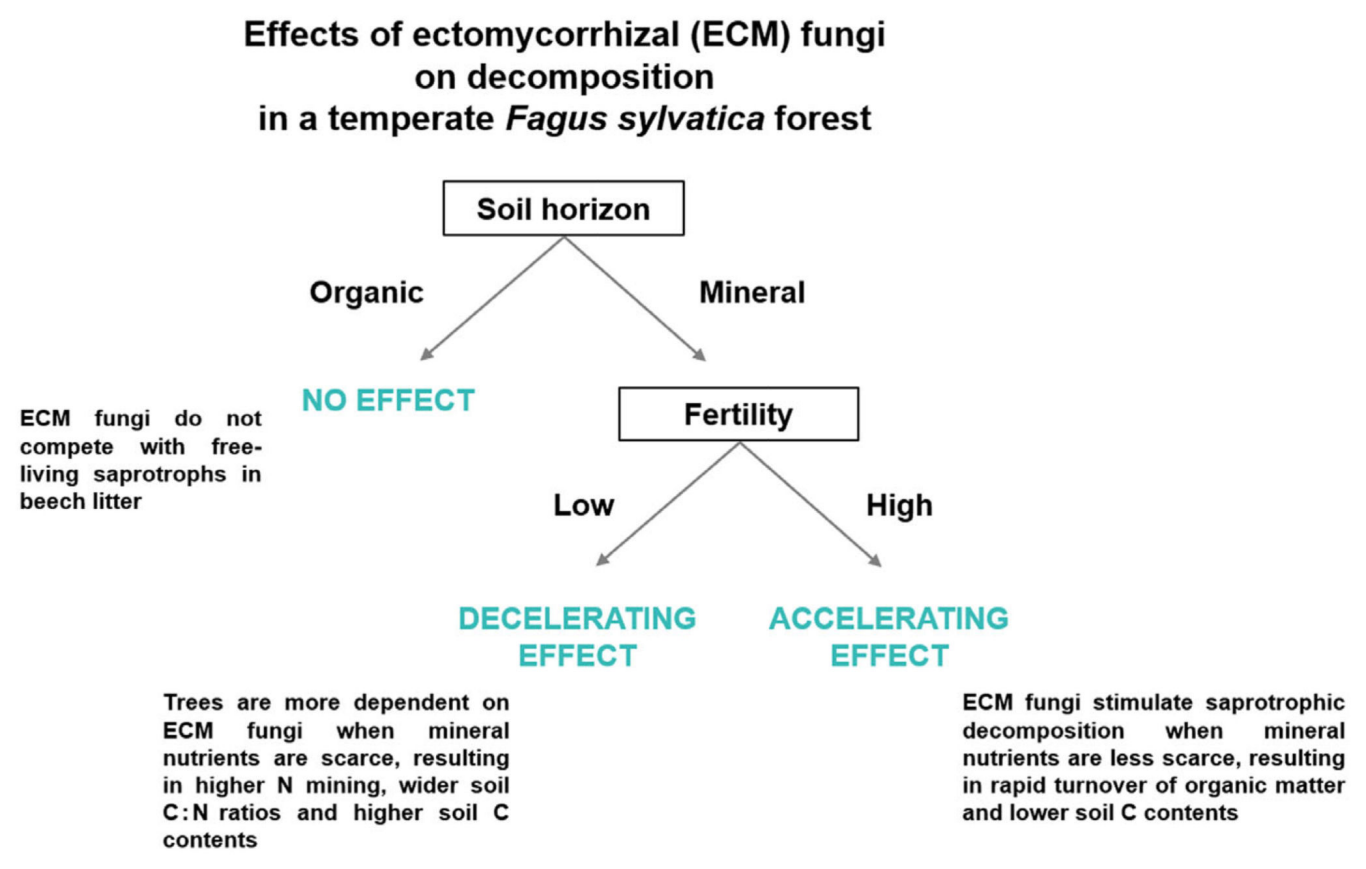
Conceptual summary on the effects of ectomycorrhizal fungi on litter and soil organic matter decomposition and soil carbon (C) and (N) dynamics along a temperate forest fertility gradient dominated by *Fagus sylvatica*.

Taken together, effects of ECM fungi on decomposition and soil C and N dynamics in the studied mountain forest of *F*. *sylvatica* depend on soil fertility and the specific soil horizon. In the organic horizon, we did not find evidence for an altered litter decomposition by ECM fungi (Fig. 8), possibly because competition between ECM fungi and saprotrophs for N is reduced when litter quality is relatively high. However, we found compelling evidence for a deceleration of C losses and decomposition in mineral soils of lower fertility. An ecological explanation may be that trees depend more strongly on their ECM symbionts when the availability of nutrients in mineral forms is low (Fig. 8; Table 1). Consequently, forests establish an organic nutrient economy characterized by more pronounced ECM mining for N and other SOM-bound nutrients (Phillips *et al*., 2013; Lang *et al*., 2016). As investment into ECM symbionts is costly, standing tree biomass and productivity are lower, and because of selective N mining, soil C : N ratios increase and soil C contents are higher under less fertile conditions. However, under conditions of high fertility, we show that ECM fungi may stimulate saprotrophic decomposition, potentially increasing turnover of N and other nutrients (Fig. 8). This indicates a rapid turnover of SOM by free-living saprotrophs and rapid cycling of inorganic nutrients (Phillips *et al*., 2013; Lang *et al*., 2016) under more fertile conditions, coinciding with a higher standing tree biomass and lower soil C : N ratios and soil C contents. We conclude that soil fertility is a key factor determining whether ECM fungi accelerate or decelerate SOM decomposition. The suppressed decomposition at low fertility reinforces nutrient limitation, while the accelerated decomposition in nutrient-rich soils fosters forest fertility. Consequently, our results suggest a positive feedback mechanism between fertility, stand productivity, and soil C and N dynamics that is mediated to a large extent by ECM fungi.

## Supplementary Material

Fig. S1, Fig. S2, Fig. S3, Fig. S4, Table S1, Table S2

Table S3

## Figures and Tables

**Table 1:** Stand and soil properties (mean ± SE) at three fertility levels (low, medium and high) in a temperate *Fagus sylvatica* forest.

Variable	Low	Medium	High
*Stand properties*			
Standing tree biomass (m^3^ ha^−1^)	373 ± 26	557 ± 70	751 ± 35
Fine root biomass (g m^−2^)	205 ± 30	243 ± 20	310 ± 46
Litter fall (g m^−2^)	205 ± 8	261 ± 17	280 ± 32
*Organic layer properties* *			
Carbon stocks (kg m^−2^)	1.3 ± 0.2	1.4 ± 0.2	0.5 ± 0.1
Carbon to nitrogen ratio (−)	23.5 ± 0.7	24.1 ± 0.8	25.6 ± 1.2
Layer thickness (cm)	7.7 ± 1.0	5.4 ± 1.7	3.2 ± 0.6
Mean residence time (yr)	11.0 ± 1.5	11.8 ± 1.0	4.0 ± 0.4
*Soil organic matter properties* **			
Organic carbon content (g g^−1^)	16.1 ± 1.1	14.8 ± 0.8	8.0 ± 0.8
Carbon to nitrogen ratio (−)	15.6 ± 0.3	15.2 ± 0.3	14.5 ± 0.1
*Soil nutrient stocks* ***			
Calcium, Ca (g m^−2^)	6.6	13.6	14.8
Magnesium, Mg (g m^−2^)	2.5	6.0	14.6
Potassium, K (g m^−2^)	2.1	6.1	14.0
Phosphorus, P (mg m^−2^)	269	554	627
Manganese, Mn (mg m^−2^)	312	566	1815

Data modified after Gorfer *et al*. (2021) and Mayer *et al*. (2021). No SE given as data derived from single soil pits.

* Litter layer (O_i_, O_e_) and humus layer if present (O_a_).

** Top mineral soil 0 to 10cm depth.

*** Mineral soil 0 to max. 50 cm depth.

**Table 2:** Test statistics on the effects of tree girdling, fertility and their interaction on soil variables measured along a temperate forest fertility gradient dominated by *Fagus sylvatica*.

Variable	Girdling		Fertility		Girdling x Fertility	
	*F*_df_	*P*	*F*_df_	*P*	*F*_df_	*P*
*Carbon fluxes*						
Soil CO_2_ efflux (imol m^−2^s^−1^)	5.17_1,26_	**0.031**	2.15_2,26_	0.137	3.89_2,26_	**0.033**
Microbial respiration (μg C g^−1^C d^−1^)	0.60_1,26_	0.446	0.52_2,26_	0.600	3.68_2,26_	**0.039**
*Substrate decomposition*						
Litter – remaining C mass (%)	0.04_1,18_	0.848	6.80_2,18_	**0.006**	0.04_2,18_	0.965
Red tea – remaining dry mass (%)	7.52_1,23_	**0.012**	5.44_2,23_	**0.012**	2.36_2,23_	0.117
Green tea – remaining dry mass (%)	1.76_1,22_	0.198	8.65_2,22_	**0.002**	0.73_2,22_	0.492
*Nitrogen form*						
Total dissolved nitrogen (mg N g^−1^ C)	4.701,26	**0.040**	4.95_2,26_	**0.015**	0.33_2,26_	0.722
Nitrate (μg N g^−1^ C)	25.0_1,26_	**< 0.001**	6.59_2,26_	**0.005**	2.17_2,26_	0.134
Ammonium (μg N g^−1^ C)	6.40_1,26_	**0.018**	10.8_2,26_	**< 0.001**	1.01_2,26_	0.377
*Soil microclimate*						
Temperature (°C)	0.50_1,26_	0.487	3.12_2,26_	0.061	0.25_2,26_	0.782
Moisture (vol.%)	< 0.01_1,26_	0.985	14.0_2,26_	**< 0.001**	2.93_2,26_	0.071
*Fungal lifestyle (relative abundance)*						
Ectomycorrhizal fungi	0.08_1,26_	0.782	1.17_2,26_	0.325	0.98_2,26_	0.388
Saprotrophic ascomycetes	0.10_1,26_	0.749	0.53_2,26_	0.596	7.04_2,26_	**0.004**
Saprotrophic basidiomycetes	2.83_1,26_	0.105	7.74_2,26_	**0.002**	2.36_2,26_	0.115
Other saprotrophic fungi	3.96_1,26_	0.057	0.15_2,26_	0.860	3.01_2,26_	0.067
Pathogenic fungi	0.12_1,26_	0.735	0.61_2,26_	0.549	0.58_2,26_	0.569
Other symbiotic fungi	1.66_1,26_	0.209	4.46_1,26_	**0.022**	0.32_2,26_	0.728
*Exploration types (relative abundance)*						
Contact types	0.03_1,26_	0.865	2.30_2,26_	0.121	3.94_2,26_	**0.032**
Short-distance types	0.10_1,26_	0.756	0.49_2,26_	0.621	4.76_2,26_	**0.017**
Long-distance types	3.13_1,26_	0.089	2.43_2,26_	0.108	0.67_2,26_	0.520
*Ectomycorrhizal hyphal production*						
Hyphal ingrowth (–)	21.4_1,26_	**< 0.001**	10.1_2,26_	**< 0.001**	0.11_2,26_	0.892
*Fungal abundance*						
Fungal DNA (μg g^−1^ C)	0.03_2,26_	0.855	6.03_1,26_	**0.007**	3.42_2,26_	**0.048**

Effects were assessed by means of linear mixed effects models. Significant (P < 0.05) effects are highlighted in bold.

**Table 3:** Test statistics of the relationship between *in vitro* microbial respiration rates (μg C g^−1^C d^−1^) and nitrogen (forms) and relative abundances of total ectomycorrhizal fungi, ectomycorrhizal exploration types or specific ectomycorrhizal genera along a temperate forest fertility gradient dominated by *Fagus sylvatica*.

Variable	Slope	*P*	*R* ^2^
*Nitrogen form*			
Total dissolved nitrogen (mg N g^−1^ C)	(+)	**< 0.0001**	0.08
Nitrate (μg N g^−1^ C)	(+)	**0.005**	0.20
Ammonium (μg N g^−1^ C)	(−)	0.498	−0.02
*Fungal lifestyle/exploration types/genera (relative abundance)*			
Ectomycorrhizal fungi	(−)	**< 0.0001**	0.13
Contact types	(−)	**0.0446**	0.02
Short-distance types	(−)	**< 0.0001**	0.13
Long-distance types	(−)	0.2619	< 0.01
*Inocybe*	(−)	**< 0.0001**	0.09
*Sebacina*	(−)	0.2359	0.01
*Clavulina*	(−)	0.3114	0.01
*Hymenogaster*	(−)	**0.0045**	0.04
*Elaphomyces*	(−)	0.8369	< 0.01
*Tomentella*	(−)	**0.0056**	0.04
*Hygrophorus*	(−)	0.1568	0.01
*Melanogaster*	(−)	0.2835	0.01

Significant (*P* < 0.05) relations are highlighted in bold, slope directions are indicated.

## Data Availability

All data used for the analyses of this study are publicly accessible. Sequencing and associated data have been deposited at NCBI BioProjects PRJNA521677 and PRJNA895381, BioSamples SAMN12582230–SAMN12582341 and SAMN31508622–SAMN31508877, and GenBank accession nos. MK626959–MK627467.
