# Supplementary material for: Soil fertility determines whether ectomycorrhizal fungi accelerate or decelerate decomposition in a temperate forest
Source: New Phytol. Author manuscript; Available in PMC 2023 Jul 1. (PMC7614611; doi:10.1111/nph.18930)
Supplement: Fig. S1, Fig. S2, Fig. S3, Fig. S4, Table S1, Table S2 [file EMS174517-supplement-Fig__S1__Fig__S2__Fig__S3__Fig__S4__Table_S1__Table_S2.pdf]

New Phytologist

***New Phytologist* Supporting Information**

Article title: Soil fertility determines whether ectomycorrhizal fungi accelerate or decelerate decomposition in a temperate forest

Authors: Mathias Mayer, Bradley Matthews, Hans Sandén, Klaus Katzensteiner, Frank Hagedorn, Markus Gorfer, Harald Berger, Torsten W. Berger, Douglas L. Godbold, Boris Rewald

Article acceptance date: 03 April 2023

The following Supporting Information is available for this article:

Fig. S1 Layout of control and girdled plots.

Fig. S2 Girdling effects on soil temperature and moisture.

Fig. S3 Girdling effects on relative abundance of pathogenic and other symbiotic fungi.

Fig. S4 Ratios ectomycorrhizal to saprotrophic fungal relative abundances.

Table S1 Test statistics pre-girdling measurements.

Table S2 Test statistics separately for fertility levels.

Table S3 List of fungal taxonomic groups including abundance and lifestyle/exploration type.

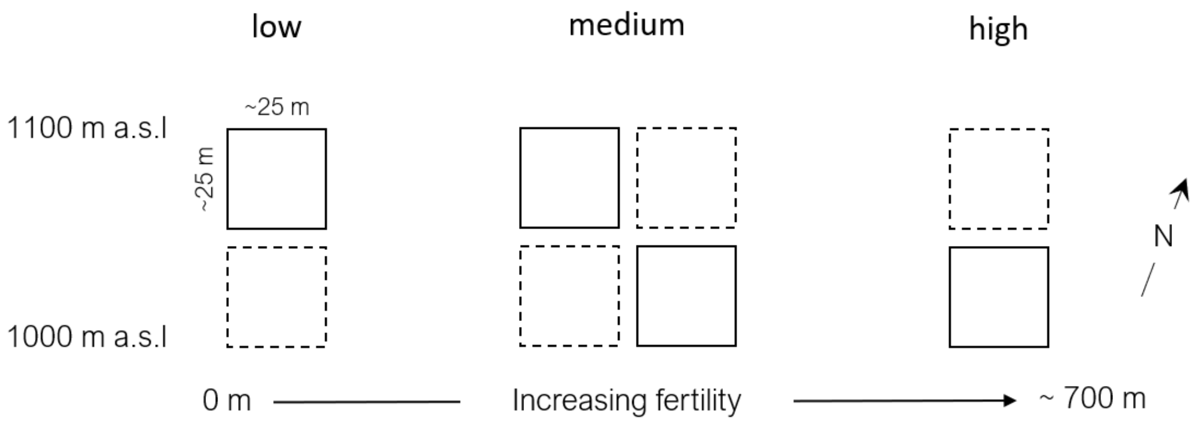

Fig. S1 Layout of control (solid line) and girdled (dashed line) plots within fertility levels (low, medium, high) along a fertility gradient in a temperate *Fagus sylvatica* forest. Cardinal N, elevation (m above sea level) and length of fertility gradient are indicated.

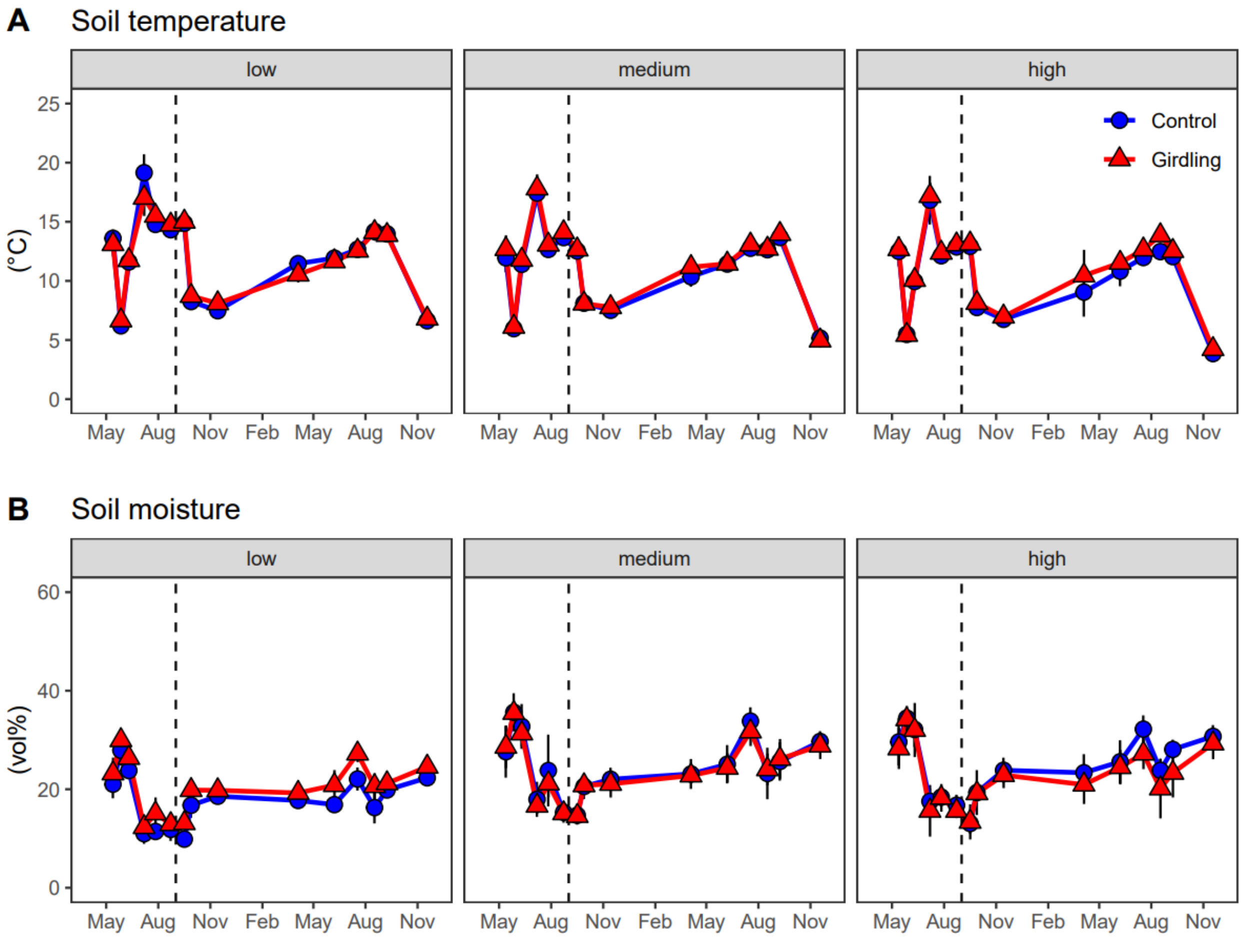

Fig. S2 Effects of tree girdling on soil temperature (**A**) and soil moisture (**B**) within fertility levels (low, medium, high) in a temperate *Fagus sylvatica* forest (mean±SE; n = 4-8). Dashed vertical lines indicate date of treatment establishment. Test statistics are given in Table 2

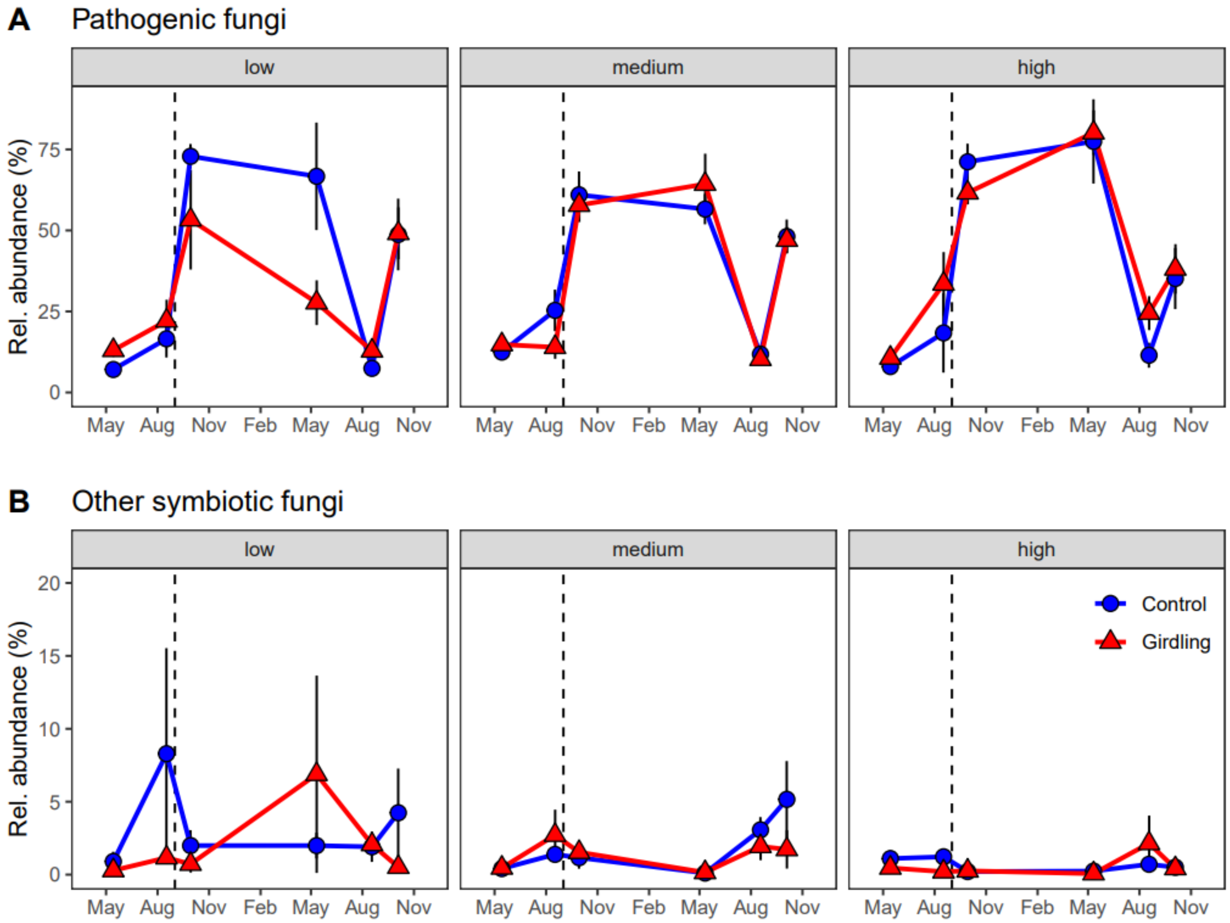

Fig. S3 Relative abundance of plant pathogenic fungi (**A**) and of other symbiotic fungi (e.g. arbuscular mycorrhizal fungi) (**B**) in mineral soils (0-10 cm) at three fertility levels (low, medium, high) and as affected by tree girdling in a temperate *Fagus sylvatica* forest (mean±SE; n = 3-8). Dashed vertical lines indicate date of girdling during the study period May 2015 to November 2016. Test statistics are given in Table 2.

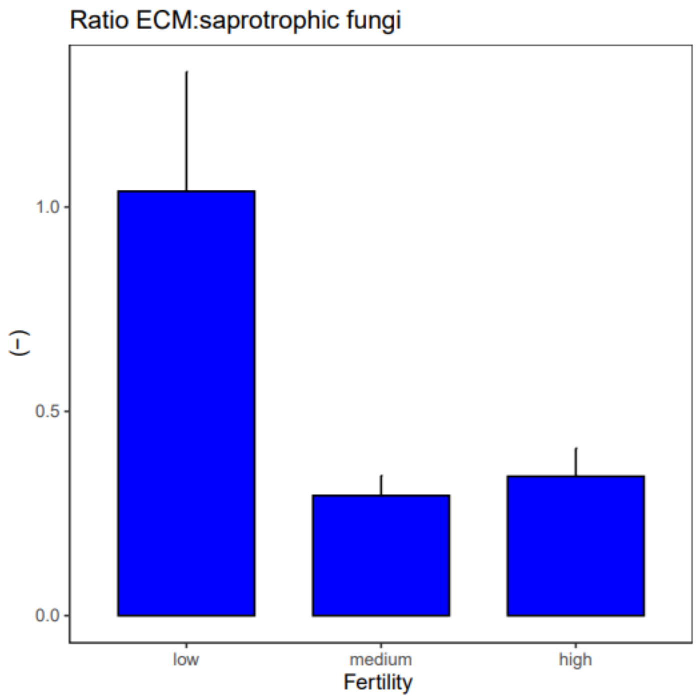

Fig. S4 Ratio between relative abundance of ectomycorrhizal (ECM) fungi and saprotrophic fungi at control plots at three fertility levels (low, medium, high) in a temperate *Fagus sylvatica* forest (mean±SE; n = 3-8).

Table S1 Test statistics comparing soil variables between control and (later) girdled plots during a pre-treatment phase (before girdling). Given are test statistics on the effects of girdling plots, fertility, and their interaction on soil variables measured in a temperate *Fagus sylvatica* forest. Effects were assessed by means of linear mixed effects models. Significant (P < 0.05) effects are highlighted in bold.

**Variable**	**Girdling**		**Fertility**		**Girdling x Fertility**	
	F_{df}	P	F_{df}	P	F_{df}	P
Carbon fluxes						
Soil CO_2 efflux (µmol m² s^{-1})	$<.01_{1,26}$	0.925	$1.29_{2,26}$	0.292	$2.06_{2,26}$	0.148
Microbial respiration (µg C $gC^{-1}d^{-1}$)	$0.29_{1,26}$	0.598	$7.05_{2,26}$	**0.004**	$0.68_{2,26}$	0.517
Substrate decomposition						
Red tea – Remaining dry mass (%)	$2.69_{1,12}$	0.127	$0.81_{2,12}$	0.469	$0.91_{2,12}$	0.430
Green tea – Remaining dry mass (%)	$1.92_{1,15}$	0.186	$4.96_{2,15}$	**0.022**	$0.33_{2,15}$	0.727
Nitrogen form						
Total dissolved nitrogen (mg N gC^{-1})	$0.22_{1,26}$	0.641	$6.33_{2,26}$	**0.006**	$2.35_{2,26}$	0.116
Soil microclimate						
Temperature (°C)	$0.17_{1,26}$	0.684	$2.04_{2,26}$	0.151	$0.10_{2,26}$	0.908
Moisture (vol.%)	$<.01_{1,26}$	0.958	$9.99_{2,26}$	**0.001**	$0.59_{2,26}$	0.560
Fungal lifestyle (relative abundance)						
Ectomycorrhizal fungi	$1.34_{1,26}$	0.258	$0.66_{2,26}$	0.527	$2.01_{2,26}$	0.154
Saprotrophic ascomycetes	$0.56_{1,26}$	0.46	$2.70_{2,26}$	0.086	$0.16_{2,26}$	0.853
Saprotrophic basidiomycetes	$0.53_{1,26}$	0.47	$6.66_{2,26}$	**0.005**	$0.44_{2,26}$	0.650
Others saprotrophic fungi	$2.19_{1,26}$	0.15	$0.27_{2,26}$	0.769	$3.23_{2,26}$	0.056
Pathogenic fungi	$0.80_{1,26}$	0.380	$0.27_{2,26}$	0.766	$2.57_{2,26}$	0.096
Other symbiotic fungi	$0.86_{1,26}$	0.436	$2.46_{2,26}$	0.129	$3.10_{2,26}$	0.062
Exploration type (relative abundance)						
Contact	$0.93_{1,26}$	0.344	$2.62_{2,26}$	0.092	$0.66_{2,26}$	0.528
Short-distance	$1.82_{,26}$	0.189	$0.65_{2,26}$	0.529	$2.36_{2,26}$	0.115
Long-distance	$3.91_{1,26}$	0.059	$1.58_{2,26}$	0.225	$0.82_{2,26}$	0.450
Fungal abundance						
Fungal DNA (µg gC^{-1})	$0.24_{1,26}$	0.626	$3.97_{2,26}$	**0.031**	$0.28_{2,26}$	0.758

Table S2 Test statistics on the effects of tree girdling (G), sampling date (S), and their interaction on soil variables measured at three fertility levels (low, medium, high) in a temperate *Fagus sylvatica* forest. Effects were assessed by means of linear mixed effects models. Significant (P < 0.05) effects are highlighted in bold.

Variable	Low fertility			Medium fertility			High fertility		
	G	S	G x S	G	S	G x S	G	S	G x S
Carbon fluxes (µmol m^2 s^{-1}; µg C $gC^{-1}d^{-1}$)									
Soil CO_2 efflux	0.2053	**<.0001**	0.3950	0.8659	**<.0001**	**0.0248**	**0.0128**	**<.0001**	**0.0001**
Microbial resp.	**0.0073**	**0.0004**	0.2809	0.9948	**<.0001**	0.0771	0.5878	0.2440	**0.0219**
Nitrogen form (mg N gC^{-1})									
Total diss. nitrogen	**0.0159**	**0.0069**	0.2331	0.2358	**0.0186**	0.0965	0.9278	**0.0005**	0.3244
Fungal lifestyle (relative abundance)									
Ectomycorrhizal	0.1744	**0.0001**	0.8778	0.1081	**<.0001**	0.1824	0.8391	**<.0001**	0.3690
Saprotr. ascom.	**0.0407**	0.7147	0.7946	0.7424	**<.0001**	0.7944	**0.0229**	**0.0011**	0.1420
Saprotr. basidiom.	**0.0497**	**0.0400**	0.7112	0.8533	0.0734	0.1676	0.1900	**0.0004**	0.5683
Other saprotrophic	0.6046	**0.0044**	0.4450	0.1864	**<.0001**	0.5686	**0.0170**	**0.0008**	0.1631
Pathogenic	0.2464	**0.0001**	0.0916	0.4319	**<.0001**	0.8207	0.6823	**<.0001**	0.5322
Other symbiotic	0.3650	0.3928	0.2883	0.2441	**0.0005**	0.1099	0.9881	0.1377	0.8284
Exploration type (relative abundance)									
Contact	0.1647	**0.0089**	0.1659	0.4690	**<.0001**	0.2827	**0.0332**	0.0635	0.3504
Short-distance	**0.0140**	**0.0002**	**0.0317**	0.4033	**<.0001**	0.0870	0.1589	**0.0002**	0.3230
Long-distance	0.7192	0.6975	0.3162	0.1151	0.0732	0.2305	0.1192	**0.0112**	0.2233

Table S3 List of fungal taxonomic groups including abundance and lifestyle/exploration type detected on a fertility gradient in a mountain forest of European beech (see separate file).
