# Supplementary material for: Soil fertility determines whether ectomycorrhizal fungi accelerate or decelerate decomposition in a temperate forest
Source: New Phytol. Author manuscript; Available in PMC 2023 Jul 1. (PMC7614611; doi:10.1111/nph.18930)
Supplement: Table S3 [file EMS174517-supplement-Table_S3.pdf]

***New Phytologist* Supporting Information**

Article title: Soil fertility determines whether ectomycorrhizal fungi accelerate or decelerate decomposition in a temperate forest

Authors: Mathias Mayer, Bradley Matthews, Hans Sandén, Klaus Katzensteiner, Frank Hagedorn, Markus Gorfer, Harald Berger, Torsten W. Berger, Douglas L. Godbold, Boris Rewald

Article acceptance date: 03 April 2023

Table S3 List of fungal taxonomic groups including abundance and lifestyle/exploration type.

Genus	Relative abundance (%)	Lifestyle/guild	ExpLong-distanceorationtype
Neonectria	20,3585	Pathogenic	
Ilyonectria	18,1738	Pathogenic	
Apiotrichum	6,8951	Saprotrophic basidiomycetes	
Mortierella	5,6152	Other saprotrophic	
Inocybe	5,3653	Ectomycorrhizal	Short-distance
Trichoderma	2,6230	Saprotrophic ascomycetes	
Dactylonectria	2,3613	Pathogenic	
Volutella	2,0144	Pathogenic	
Leotiomycetes	1,9330	Unknown	
Clavulina	1,6461	Ectomycorrhizal	Contact
Saitozyma	1,5084	Saprotrophic basidiomycetes	
Sebacina	1,3942	Ectomycorrhizal	Short-distance
Exophiala	1,1041	Saprotrophic ascomycetes	
Sebacinaceae	1,0664	Other symbiotic	Short-distance
Sordariomycetes	1,0627	Unknown	
Fungus	0,9767	Unknown	
Geminibasidium	0,9701	Saprotrophic basidiomycetes	
Tetracladium	0,9368	Saprotrophic ascomycetes	
Fusarium	0,9036	Pathogenic	
Nectriaceae	0,8068	Pathogenic	
Pezizomycotina	0,7939	Unknown	
Hyaloscyphaceae	0,7871	Saprotrophic ascomycetes	
Hymenogaster	0,7600	Ectomycorrhizal	Short-distance
Elaphomyces	0,7445	Ectomycorrhizal	Short-distance
Chytridiomycetes	0,6605	Unknown	
Tomentella	0,6593	Ectomycorrhizal	Medium-distance
Oidiodendron	0,6106	Saprotrophic ascomycetes	
Fusicolla	0,5727	Pathogenic	
Sebacinales	0,5149	Other symbiotic	Contact
Bionectria	0,4932	Saprotrophic ascomycetes	
Cladophialophora	0,4919	Saprotrophic ascomycetes	
Microbotryomycetes	0,4805	Unknown	
Metarhizium	0,4648	Saprotrophic ascomycetes	
Solicoccozyma	0,3917	Saprotrophic basidiomycetes	
Helotiales	0,3904	Unknown	
Melanogaster	0,3851	Ectomycorrhizal	Long-distance
Herpotrichiellaceae	0,3834	Saprotrophic ascomycetes	
Schizothecium	0,3833	Saprotrophic ascomycetes	
Agaricales	0,3731	Unknown	
Humicola	0,3544	Saprotrophic ascomycetes	
Cryptocoryneum	0,3253	Saprotrophic ascomycetes	
Archaeorhizomycetes	0,3122	Saprotrophic ascomycetes	
Microdochiella	0,3088	Saprotrophic ascomycetes	
Hygrophorus	0,3086	Ectomycorrhizal	Contact
Preussia	0,2956	Saprotrophic ascomycetes	
Sagenomella	0,2928	Saprotrophic ascomycetes	
Pleosporineae	0,2880	Unknown	
Hyaloscyphaceae_ECM	0,2789	Ectomycorrhizal	Short-distance
Penicillium	0,2622	Saprotrophic ascomycetes	
Suillellus	0,2576	Ectomycorrhizal	Long-distance
Mycena	0,2444	Saprotrophic basidiomycetes	
Lambertella	0,2438	Saprotrophic ascomycetes	
Mucor	0,2386	Other saprotrophic	
Phloeomana	0,2235	Saprotrophic basidiomycetes	
Cortinarius	0,2222	Ectomycorrhizal	Medium-distance

Epicoccum	0,2165	Saprotrophic ascomycetes	
Orbiliales	0,2055	Saprotrophic ascomycetes	
Pleosporales	0,2045	Unknown	
Apodus	0,2033	Saprotrophic ascomycetes	
Chrysozymaceae	0,2017	Saprotrophic basidiomycetes	
Agaricostilbales	0,1782	Saprotrophic basidiomycetes	
Saccharomycetales	0,1771	Saprotrophic ascomycetes	
Entoloma	0,1669	Saprotrophic basidiomycetes	
Cordyceps	0,1658	Saprotrophic ascomycetes	
Lasiosphaeriaceae	0,1574	Saprotrophic ascomycetes	
Pleotrichocladium	0,1464	Pathogenic	
Coniochaeta	0,1429	Saprotrophic ascomycetes	
Rhizophydiales	0,1394	Other saprotrophic	
Sordariales	0,1298	Saprotrophic ascomycetes	
Cadophora	0,1274	Saprotrophic ascomycetes	
Basidiomycota	0,1269	Unknown	
Brunneochlamydosporium	0,1256	Pathogenic	
Mrakia	0,1240	Saprotrophic basidiomycetes	
Hymenochaetaceae	0,1208	Saprotrophic basidiomycetes	
Aspergillus	0,1081	Saprotrophic ascomycetes	
Metapochonia	0,1068	Saprotrophic ascomycetes	
Cosmospora	0,0943	Saprotrophic ascomycetes	
Saccharomycopsis	0,0929	Saprotrophic ascomycetes	
Atheliaceae	0,0922	Unknown	
Hypocreomycetidae	0,0913	Unknown	
Thielaviopsis	0,0905	Pathogenic	
Cylindrodendrum	0,0892	Pathogenic	
Minutisphaera	0,0847	Saprotrophic ascomycetes	
Cephalotrichum	0,0737	Saprotrophic ascomycetes	
Pezicula	0,0734	Saprotrophic ascomycetes	
Dikarya	0,0730	Unknown	
Xylariales	0,0709	Saprotrophic ascomycetes	
Rhizophydium	0,0634	Other saprotrophic	
Papiliotrema	0,0598	Saprotrophic basidiomycetes	
Alatospora	0,0597	Saprotrophic ascomycetes	
Pyronemataceae	0,0573	Unknown	
Pseudeurotium	0,0547	Saprotrophic ascomycetes	
Chalara	0,0536	Saprotrophic ascomycetes	
Ganoderma	0,0522	Saprotrophic basidiomycetes	
Clavulinaceae	0,0519	Ectomycorrhizal	Contact
Wardomyces	0,0495	Saprotrophic ascomycetes	
Dothideomycetes	0,0487	Unknown	
Polyphilus	0,0483	Saprotrophic ascomycetes	
Cyphellophora	0,0482	Saprotrophic ascomycetes	
Ijuhya	0,0480	Saprotrophic ascomycetes	
Paraleptosphaeria	0,0472	Pathogenic	
Clavariaceae	0,0461	Saprotrophic basidiomycetes	
Helotiaceae	0,0442	Saprotrophic ascomycetes	
Agaricomycetes	0,0433	Unknown	
Xylariaceae	0,0414	Saprotrophic ascomycetes	
Lophiostoma	0,0410	Saprotrophic ascomycetes	
Hypocreales	0,0403	Saprotrophic ascomycetes	
Pezizales	0,0398	Unknown	
Scutellinia	0,0383	Saprotrophic ascomycetes	
Chaetosphaeria	0,0373	Saprotrophic ascomycetes	
Geomyces	0,0372	Saprotrophic ascomycetes	

Chaetothyriomycetidae	0,0356	Saprotrophic ascomycetes	
Chaetomiaceae	0,0342	Saprotrophic ascomycetes	
Glomeraceae	0,0334	Other symbiotic	
Dictyosporium	0,0332	Saprotrophic ascomycetes	
Xylaria	0,0331	Saprotrophic ascomycetes	
Tremellomycetes	0,0330	Unknown	
Eurotiomycetes	0,0327	Saprotrophic ascomycetes	
Ascomycota	0,0318	Unknown	
Rhexocercosporidium	0,0316	Pathogenic	
Melanommataceae	0,0315	Pathogenic	
Sordariomycetidae	0,0310	Unknown	
Clonostachys	0,0303	Saprotrophic ascomycetes	
Leotiaceae	0,0292	Saprotrophic ascomycetes	
Leohumicola	0,0291	Saprotrophic ascomycetes	
Plectosphaerellaceae	0,0289	Pathogenic	
Orbiliaceae	0,0289	Saprotrophic ascomycetes	
Archaeorhizomyces	0,0276	Saprotrophic ascomycetes	
Idriella	0,0274	Saprotrophic ascomycetes	
Cytospora	0,0251	Pathogenic	
Microascaceae	0,0251	Saprotrophic ascomycetes	
Phialemoniopsis	0,0250	Saprotrophic ascomycetes	
Chaetothyriales	0,0249	Saprotrophic ascomycetes	
Hypomyces	0,0244	Saprotrophic ascomycetes	
Acremonium_Bionectriaceae	0,0244	Saprotrophic ascomycetes	
Pyxidiophoraceae	0,0242	Saprotrophic ascomycetes	
Octaviania	0,0242	Ectomycorrhizal	Long-distance
Vanrija	0,0240	Saprotrophic basidiomycetes	
Polyporales	0,0236	Unknown	
Glomerales	0,0235	Other symbiotic	
Chionosphaeraceae	0,0233	Saprotrophic basidiomycetes	
Capronia	0,0227	Saprotrophic ascomycetes	
Meliniomyces	0,0223	Saprotrophic ascomycetes	
Spizellomycetales	0,0222	Other saprotrophic	
Eurotiales	0,0221	Saprotrophic ascomycetes	
Operculomyces	0,0219	Other saprotrophic	
Leucoagaricus	0,0214	Saprotrophic basidiomycetes	
Rhizoctonia	0,0209	Pathogenic	
Podospora	0,0186	Saprotrophic ascomycetes	
Cenococcum	0,0183	Ectomycorrhizal	Short-distance
Heterocephalacria	0,0183	Saprotrophic basidiomycetes	
Helvellosebacina	0,0180	Saprotrophic basidiomycetes	
Cladosporium	0,0179	Saprotrophic ascomycetes	
Hyphodontia	0,0174	Saprotrophic basidiomycetes	
Phaeococcomyces	0,0174	Saprotrophic ascomycetes	
Pseudogymnoascus	0,0168	Saprotrophic ascomycetes	
Memnoniella	0,0163	Saprotrophic ascomycetes	
Glomeromycota	0,0161	Other symbiotic	
Tricholomataceae	0,0160	Unknown	
Phyllozyma	0,0159	Saprotrophic basidiomycetes	
Xenopolyscytalum	0,0159	Saprotrophic ascomycetes	
Pseudopyrenochaeta	0,0153	Pathogenic	
Bartaliniaceae	0,0148	Saprotrophic ascomycetes	
Coprinellus	0,0147	Saprotrophic basidiomycetes	
Striatibotrys	0,0144	Saprotrophic ascomycetes	
Hebeloma	0,0144	Ectomycorrhizal	Short-distance
Tuber	0,0129	Ectomycorrhizal	Short-distance

Hysterangium	0,0129	Ectomycorrhizal	Medium-distance
Tolypocladium	0,0124	Saprotrophic ascomycetes	
Trichosporonaceae	0,0117	Saprotrophic basidiomycetes	
Monosporascus	0,0115	Pathogenic	
Lecanicillium	0,0113	Saprotrophic ascomycetes	
Nigrograna	0,0113	Saprotrophic ascomycetes	
Lopadostoma	0,0110	Saprotrophic ascomycetes	
Flagelloscypha	0,0107	Saprotrophic basidiomycetes	
Tarzetta	0,0107	Ectomycorrhizal	Contact
Hymenoscyphus	0,0104	Saprotrophic ascomycetes	
Hyaloriaceae	0,0101	Saprotrophic basidiomycetes	
Basidioascus	0,0100	Saprotrophic basidiomycetes	
Malassezia	0,0097	Saprotrophic basidiomycetes	
Thelonectria	0,0096	Pathogenic	
Phellodon	0,0095	Ectomycorrhizal	Medium-distance
Leptodontidium	0,0095	Saprotrophic ascomycetes	
Nemania	0,0094	Saprotrophic ascomycetes	
Lycoperdon	0,0094	Saprotrophic basidiomycetes	
Curvularia	0,0094	Pathogenic	
Aureobasidium	0,0094	Saprotrophic ascomycetes	
Phaeosphaeriaceae	0,0092	Pathogenic	
Candida	0,0089	Saprotrophic ascomycetes	
Microthyriaceae	0,0089	Unknown	
Sistotrema_ECM	0,0089	Ectomycorrhizal	Unknown
Atractospora	0,0088	Saprotrophic ascomycetes	
Gymnostellatospora	0,0088	Saprotrophic ascomycetes	
Tausonia	0,0085	Saprotrophic basidiomycetes	
Pseudeurotiaceae	0,0084	Saprotrophic ascomycetes	
Tubeufiaceae	0,0084	Saprotrophic ascomycetes	
Capnodiales	0,0083	Saprotrophic ascomycetes	
Diversispora	0,0080	Other symbiotic	
Spadicoides	0,0079	Saprotrophic ascomycetes	
Chaetosphaeriaceae	0,0079	Saprotrophic ascomycetes	
Gautieria	0,0078	Ectomycorrhizal	Medium-distance
Xylodon	0,0077	Saprotrophic basidiomycetes	
Mycenella	0,0076	Saprotrophic basidiomycetes	
Botryosphaeriaceae	0,0076	Pathogenic	
Cystofilobasidium	0,0073	Saprotrophic basidiomycetes	
Cephalothecaceae	0,0072	Saprotrophic ascomycetes	
Ceratobasidiaceae	0,0071	Pathogenic	
Agaricomycotina	0,0071	Unknown	
Minimelanolocus	0,0070	Saprotrophic ascomycetes	
Scopuloides	0,0070	Saprotrophic basidiomycetes	
Piloderma	0,0070	Ectomycorrhizal	Short-distance
Darksidea	0,0070	Saprotrophic ascomycetes	
Pestalotiopsidaceae	0,0068	Pathogenic	
Clavaria	0,0068	Saprotrophic basidiomycetes	
Basidiobolus	0,0068	Unknown	
Verruconis	0,0066	Saprotrophic ascomycetes	
Dictyosporiaceae	0,0065	Saprotrophic ascomycetes	
Cladobotryum	0,0064	Saprotrophic ascomycetes	
Psathyrella	0,0064	Saprotrophic basidiomycetes	
Lophiotrema	0,0064	Saprotrophic ascomycetes	
Leucosporidium	0,0063	Saprotrophic basidiomycetes	
Sakaguchia	0,0062	Saprotrophic basidiomycetes	
Neocucurbitaria	0,0059	Saprotrophic ascomycetes	

Glomerellaceae	0,0058	Pathogenic	
Phaeotheca	0,0058	Saprotrophic ascomycetes	
Umbelopsis	0,0058	Other saprotrophic	
Eurotiomycetidae	0,0058	Saprotrophic ascomycetes	
Lactarius	0,0055	Ectomycorrhizal	Medium-distance
Thelebolus	0,0054	Saprotrophic ascomycetes	
Agaricomycetidae	0,0052	Unknown	
Discosia	0,0051	Saprotrophic ascomycetes	
Glomus	0,0051	Other symbiotic	
Mucoromycotina	0,0051	Other saprotrophic	
Didymosphaeriaceae	0,0051	Saprotrophic ascomycetes	
Apioperdon	0,0050	Saprotrophic basidiomycetes	
Cordana	0,0049	Saprotrophic ascomycetes	
Pachyphlodes	0,0048	Ectomycorrhizal	Short-distance
Membranomyces	0,0047	Ectomycorrhizal	Contact
Sporocadaceae	0,0047	Unknown	
Mortierellaceae	0,0047	Other saprotrophic	
Trichocladium	0,0045	Saprotrophic ascomycetes	
Roesleria	0,0044	Pathogenic	
Myxotrichaceae	0,0044	Saprotrophic ascomycetes	
Stictidaceae	0,0044	Other symbiotic	
Zoopagomycota	0,0044	Unknown	
Trametes	0,0043	Saprotrophic basidiomycetes	
Pleosporomycetidae	0,0042	Unknown	
Microdochium	0,0040	Pathogenic	
Venturiales	0,0040	Saprotrophic ascomycetes	
Trechispora	0,0040	Saprotrophic basidiomycetes	
Pyrenochaetopsis	0,0039	Saprotrophic ascomycetes	
Massarineae	0,0038	Unknown	
Valsaceae	0,0037	Pathogenic	
Neohendersonia	0,0037	Saprotrophic ascomycetes	
Hyaloscypha	0,0037	Other symbiotic	Unknown
Pleurotheciaceae	0,0035	Saprotrophic ascomycetes	
Jobellisia	0,0035	Saprotrophic ascomycetes	
Hypoxylon	0,0034	Saprotrophic ascomycetes	
Tremellales	0,0034	Saprotrophic basidiomycetes	
Acephala	0,0034	Saprotrophic ascomycetes	
Melanomma	0,0033	Pathogenic	
Knufia	0,0032	Saprotrophic ascomycetes	
Veronaea	0,0032	Saprotrophic ascomycetes	
Ovicillium	0,0031	Saprotrophic ascomycetes	
Rigidoporus	0,0030	Saprotrophic basidiomycetes	
Geniculifera	0,0030	Saprotrophic ascomycetes	
Thelephoraceae	0,0030	Ectomycorrhizal	Contact
Clitopilus	0,0030	Saprotrophic basidiomycetes	
Hyalopeziza	0,0030	Saprotrophic ascomycetes	
Montagnula	0,0029	Saprotrophic ascomycetes	
Venturia	0,0029	Pathogenic	
Paraphaeosphaeria	0,0029	Saprotrophic ascomycetes	
Heterobasidion	0,0028	Saprotrophic basidiomycetes	
Lentithecium	0,0027	Saprotrophic ascomycetes	
Neohelicomyces	0,0027	Saprotrophic ascomycetes	
Pseudotomentella	0,0027	Ectomycorrhizal	Medium-distance
Phacidiella	0,0027	Pathogenic	
Archaeospora	0,0026	Other symbiotic	
Rhizophlyctis	0,0026	Other saprotrophic	

Phragmocephala	0,0025	Saprotrophic ascomycetes	
Debaryomyces	0,0025	Saprotrophic ascomycetes	
Cystobasidiomycetes	0,0025	Unknown	
Atractium	0,0024	Pathogenic	
Arthrinium	0,0023	Saprotrophic ascomycetes	
Sporoschisma	0,0023	Saprotrophic ascomycetes	
Kockovaella	0,0023	Saprotrophic basidiomycetes	
Itersonilia	0,0022	Pathogenic	
Lecanoromycetes	0,0022	Other symbiotic	
Dacrymycetaceae	0,0022	Saprotrophic basidiomycetes	
Hymenopellis	0,0022	Saprotrophic basidiomycetes	
Tricharina	0,0022	Saprotrophic ascomycetes	
Alternaria	0,0021	Pathogenic	
Hypholoma	0,0021	Saprotrophic basidiomycetes	
Fenestella	0,0021	Saprotrophic ascomycetes	
Dermateaceae	0,0020	Saprotrophic ascomycetes	
Amblyosporium	0,0019	Saprotrophic ascomycetes	
Cladorrhinum	0,0019	Saprotrophic ascomycetes	
Coprinopsis	0,0018	Saprotrophic basidiomycetes	
Flagellospora	0,0017	Saprotrophic ascomycetes	
Suillus	0,0016	Ectomycorrhizal	Long-distance
Ochroconis	0,0016	Saprotrophic ascomycetes	
Ophiosphaerella	0,0016	Pathogenic	
Glomerellales	0,0016	Pathogenic	
Cyphellophoraceae	0,0016	Saprotrophic ascomycetes	
Vibrisseaceae	0,0016	Saprotrophic ascomycetes	
Crepidotus	0,0015	Saprotrophic basidiomycetes	
Lasionectria	0,0015	Saprotrophic ascomycetes	
Martininia	0,0015	Pathogenic	
Gonytrichum	0,0015	Saprotrophic ascomycetes	
Plectosphaerella	0,0015	Pathogenic	
Microcera	0,0015	Pathogenic	
Lindgomycetaceae	0,0015	Saprotrophic ascomycetes	
Henningsomyces	0,0014	Saprotrophic basidiomycetes	
Chaetomium	0,0014	Saprotrophic ascomycetes	
Meripilaceae	0,0014	Saprotrophic basidiomycetes	
Massarinaceae	0,0014	Saprotrophic ascomycetes	
Aspergillaceae	0,0013	Saprotrophic ascomycetes	
Neurospora	0,0013	Saprotrophic ascomycetes	
Pseudopithomyces	0,0013	Pathogenic	
Lipomyces	0,0013	Saprotrophic ascomycetes	
Phacidium	0,0013	Pathogenic	
Chaetothyriaceae	0,0013	Saprotrophic ascomycetes	
Serendipita	0,0013	Saprotrophic basidiomycetes	
Lophiostomataceae	0,0012	Saprotrophic ascomycetes	
Serendipitaceae	0,0012	Other symbiotic	Contact
Holtermanniella	0,0012	Saprotrophic basidiomycetes	
Udeniozyma	0,0012	Saprotrophic basidiomycetes	
Phallomycetidae	0,0011	Unknown	
Hyphodermella	0,0011	Saprotrophic basidiomycetes	
Gloeohypochnicium	0,0011	Saprotrophic basidiomycetes	
Leptosphaeria	0,0010	Pathogenic	
Parafenestella	0,0010	Saprotrophic ascomycetes	
Pyrenophora	0,0010	Pathogenic	
Reticulascus	0,0010	Pathogenic	
Dothistroma	0,0009	Pathogenic	

Tilletia	0,0009	Pathogenic	
Echinoderma	0,0009	Saprotrophic basidiomycetes	
Rhodosporidiobolus	0,0009	Saprotrophic basidiomycetes	
Burgoa	0,0008	Saprotrophic basidiomycetes	
Rhizopus	0,0008	Other saprotrophic	
Tangerinosporium	0,0008	Saprotrophic ascomycetes	
Auriculariales	0,0008	Saprotrophic basidiomycetes	
Testudinaceae	0,0008	Unknown	
Goffeauzyma	0,0008	Saprotrophic basidiomycetes	
Leotiomycetidae	0,0008	Unknown	
Pseudoanungitea	0,0008	Pathogenic	
Neobulgaria	0,0007	Saprotrophic ascomycetes	
Conocybe	0,0007	Saprotrophic basidiomycetes	
Cortinariaceae	0,0007	Ectomycorrhizal	Medium-distance
Diatrypaceae	0,0007	Saprotrophic ascomycetes	
Pouzarella	0,0006	Saprotrophic basidiomycetes	
Kretzschmaria	0,0006	Pathogenic	
Claroideoglomus	0,0006	Other symbiotic	
Marasmius	0,0005	Saprotrophic basidiomycetes	
Monilinia	0,0005	Pathogenic	
Mycocentrospora	0,0005	Pathogenic	
Rutstroemiaceae	0,0005	Saprotrophic ascomycetes	
Pleurophoma	0,0005	Saprotrophic ascomycetes	
Hydnobolites	0,0005	Ectomycorrhizal	Short-distance
Hawksworthiana	0,0005	Pathogenic	
Antarctomyces	0,0004	Saprotrophic ascomycetes	
Leucocoprinus	0,0004	Saprotrophic basidiomycetes	
Rhytismataceae	0,0004	Pathogenic	
Geastrum	0,0004	Saprotrophic basidiomycetes	
Gliomastix	0,0004	Saprotrophic ascomycetes	
Thyridariaceae	0,0003	Saprotrophic ascomycetes	
Tricellula	0,0003	Saprotrophic ascomycetes	
Neodevriesia	0,0003	Saprotrophic ascomycetes	
Dictyochaeta	0,0003	Saprotrophic ascomycetes	
Pholiota	0,0003	Saprotrophic basidiomycetes	
Taphrina	0,0003	Pathogenic	
Brachysporium	0,0002	Saprotrophic ascomycetes	
Nectriopsis	0,0002	Saprotrophic ascomycetes	
Climacocystis	0,0002	Saprotrophic basidiomycetes	
Colletotrichum	0,0002	Pathogenic	
Ascobolus	0,0002	Saprotrophic ascomycetes	
Myrmecridium	0,0002	Saprotrophic ascomycetes	
Neosetophoma	0,0002	Pathogenic	
Dioszegia	0,0002	Saprotrophic basidiomycetes	
Incertomyces	0,0002	Saprotrophic ascomycetes	
Terramycetaceae	0,0002	Other saprotrophic	
Cystolepiota	0,0001	Saprotrophic basidiomycetes	
Saxophila	0,0001	Saprotrophic ascomycetes	
Leptodiscella	0,0001	Saprotrophic ascomycetes	
Tumularia	0,0001	Saprotrophic ascomycetes	
Phaeotremella	0,0001	Saprotrophic basidiomycetes	
Chaetopsis	0,0001	Saprotrophic ascomycetes	
Pucciniomycetes	0,0001	Unknown	
Macrophomina	0,0001	Pathogenic	
Neoascochyta	0,0001	Pathogenic	
Botryotrichum	0,0001	Saprotrophic ascomycetes	

Adelphella	0,0001	Saprotrophic ascomycetes	
Lachnaceae	0,0001	Saprotrophic ascomycetes	
Rhizodiscina	0,0000	Saprotrophic ascomycetes	
Amanita	0,0000	Ectomycorrhizal	Contact
